# The Novel Dual GIP and GLP‐1 Receptor Agonist Tirzepatide Attenuates Colon Cancer Development by Regulating Glucose Metabolism

**DOI:** 10.1002/advs.202411980

**Published:** 2025-03-24

**Authors:** Yikai Zhang, Yi Xie, Shenglong Xia, Xinnuo Ge, Jiaying Li, Fang Liu, Fan Jia, Shengyao Wang, Qiao Zhou, Menghan Gao, Weihuan Fang, Chao Zheng

**Affiliations:** ^1^ Department of Endocrinology The Second Affiliated Hospital School of Medicine Zhejiang University Hangzhou 310009 P. R. China; ^2^ Department of Gastroenterology The Second Affiliated Hospital School of Medicine Zhejiang University Hangzhou 310009 P. R. China; ^3^ Center for Basic and Translational Research The Second Affiliated Hospital School of Medicine Zhejiang University Hangzhou 310009 P. R. China; ^4^ MOE Key Laboratory of Macromolecule Synthesis and Functionalization of Ministry of Education Department of Polymer Science and Engineering Zhejiang University Hangzhou 310009 P. R. China; ^5^ Department of Veterinary Medicine Zhejiang University Hangzhou 310009 P. R. China

**Keywords:** anti‐colorectal cancer effect, glucose metabolism, tirzepatide

## Abstract

Colorectal cancer (CRC) is a leading cause of cancer mortality while diabetes is a recognized risk factor for CRC. Here we report that tirzepatide (TZP), a novel polypeptide/glucagon‐like peptide 1 receptor (GIPR/GLP‐1R) agonist for the treatment of diabetes, has a role in attenuating CRC growth. TZP significantly inhibited colon cancer cell proliferation promoted apoptosis in vitro and induced durable tumor regression in vivo under hyperglycemic and nonhyperglycemic conditions across multiple murine cancer models. As glucose metabolism is known to critically regulate colon cancer progression, spatial metabolomics results revealed that glucose metabolites are robustly reduced in the colon cancer regions of the TZP‐treated mice. TZP inhibited glucose uptake and destabilized hypoxia‐inducible factor‐1 alpha (HIF‐1α) with reduced expression and activity of the rate‐limiting enzymes 6‐phosphofructo‐2‐kinase/fructose‐2,6‐bisphosphatase 3 (PFKFB3) and phosphofructokinase 1 (PFK‐1). These effects contributed to the downregulation of glycolysis and the tricarboxylic acid (TCA) cycle. TZP also delayed tumor development in a patient‐derived xenograft (PDX) mouse model accompanied by HIF‐1α mediated PFKFB3‐PFK‐1 inhibition. Therefore, the study provides strong evidence that glycolysis‐blocking TZP, besides its application in treating type 2 diabetes, has the potential for preclinical studies as a therapy for colorectal cancer used either as monotherapy or in combination with other anticancer therapies.

## Graphical abstract

Tirzepatide, a dual GIP/GLP‐1 receptor agonist for diabetes, suppresses colorectal cancer growth by targeting glucose metabolism. It inhibits cancer cell proliferation, promotes apoptosis, and induces tumor regression. It reduces glucose uptake and destabilizes HIF‐1α for degradation to downregulate PFKFB3/PFK‐1 enzymes, thus contributing to the inhibition of glucose metabolism. These findings highlight its potential as a dual‐action therapy for diabetes‐associated colorectal cancer.

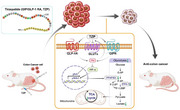

## 1 Introduction

Type 2 diabetes mellitus (T2DM) is an important risk factor for many cancers including colorectal cancer (CRC).^[^ ^1^ ^]^ A meta‐analytical study suggests that T2DM is an independent risk factor for colon cancer.^[^ ^2^ ^]^ It could increase the risk of developing CRC by 27%.^[^ ^3^ ^]^ T2DM and CRC share risk factors like age, obesity, smoking, and alcohol intake. However, the exact biological link between them is yet to be determined. Proposed mechanisms include elevated glucose and insulin levels, adipokines, chemokines, and immune function changes.^[^ ^4^ ^]^ Considering the shared risk factors of both conditions and the heightened risk of CRC in individuals with T2DM, it would be reasonable and of great interest to explore the potential of conventional type 2 diabetes therapies as an avenue for CRC prevention.

Tirzepatide (TZP) is a novel dual GIP and GLP‐1 receptor agonist (GIP/GLP‐1RA) for the treatment of T2DM. In preclinical studies, TZP showed robust improvements in glycemic control and body weight, without increased risk of hypoglycemia.^[^ ^5^ ^]^ GLP‐1 receptor agonists were reported to exhibit synergistic anti‐tumor properties.^[^ ^6^ ^]^ Liraglutide decreased the viability of LNCaP prostate cancer cells when it was combined with docetaxel,^[^ ^7^ ^]^ and also had synergistic antitumor effects with metformin on pancreatic cancer cells.^[^ ^8^ ^]^ In colon cancer cells, exenatide reduced the survival of murine colon cancer cell lines and activated pro‐apoptotic caspase 3/7.^[^ ^9^ ^]^ A recent meta‐analysis suggested that TZP may not increase the risk of cancer among individuals with T2DM.^[^ ^10^ ^]^ Whether TZP could benefit cancer therapeutics when used as an anti‐diabetic drug in diabetic patients is yet to be determined.

Cancer cells employ both conventional oxidative metabolism and glycolytic anaerobic metabolism. However, their proliferation is marked by a shift toward glycolytic metabolism even in the presence of O_2_ (Warburg effect).^[^ ^11^ ^]^ HIF‐1α, a major hypoxia‐induced transcription factor, can promote the Warburg effect by upregulating key glycolytic enzymes and glucose transporters, thereby enhancing glycolysis and lactate production in cancer cells.^[^ ^12^ ^]^ The glycolytic flux is regulated mainly through the key rate‐limiting step controlled by phosphofructokinase‐1 (PFK‐1), which catalyzes the transformation of fructose‐6‐phosphate (F‐6‐P) to fructose‐1,6‐bisphosphate (F‐1,6‐BP).^[^ ^13^ ^]^ Homodimeric bifunctional 6‐phosphofructo‐2‐kinase/fructose‐2,6‐bisphosphatase 3 (PFKFB3) phosphorylates F‐6‐P to fructose‐2,6‐bisphosphate (F‐2,6‐BP), which subsequently allosterically activates PFK‐1 and stimulates high glycolytic flux in human cancers.^[^ ^14^ ^]^ Dysregulation of metabolic pathways has been suggested to contribute to the pathogenesis of cancer, and this altered metabolism introduces metabolic liabilities that can be exploited for cancer therapy.^[^ ^15^ ^]^

In the present study, we showed that TZP attenuated the proliferation of CRC cells and delayed the growth of CRC tumors in multiple murine models with type 1 diabetes type 2 diabetes, or non‐diabetes. In spatial metabolomics, we found that TZP treatment significantly reduced the accumulation of glucose metabolic products in the tumor region. We hypothesized that TZP inhibited CRC cell proliferation by affecting glucose metabolism. In further in vitro experiments, we verified the inhibitory effect of TZP on glucose uptake across different CRC cell lines. TZP suppressed glucose uptake, and reduced expression of key glycolytic enzymes PFKFB3 and PFK‐1, thereby limiting glycolytic and tricarboxylic acid (TCA) flux, leading to energy deprivation and anti‐tumor effects. Furthermore, we found that TZP destabilized HIF‐1α by promoting proteasomal degradation with subsequent downregulation of PFKFB3. In the patient‐derived xenograft (PDX) model, we observed that the inhibitory effect of TZP on tumors was accompanied by lower expression of HIF‐1α, PFKFB3, and PFK‐1 in the tissues. Thus, our study not only suggests a therapeutic implication of TZP in obesity‐ and T2DM‐associated CRC but also provides insight into primary mechanisms of anti‐CRC effects by downregulating glucose metabolism.

## 2 Results

### 2.1 TZP Inhibited Proliferation and Migration of CRC Cells and Induced Apoptosis Regardless of High‐Glucose Stress In Vitro

To our knowledge, no prior studies have reported the effective concentration range of TZP in cancer cell models.^[^ ^16^ ^]^ Therefore, we conducted dose‐response experiments to determine the minimal concentration required to observe significant anti‐proliferative effects in CRC cell lines. TZP showed a dose‐dependent inhibition of the proliferation of colonic cancer MC38 cells from 5 up to 50 µM without apparent toxic effect on the mouse embryonic hepatocytes line (BNL CL.2) (Figure S1, Supporting Information). In human cell lines HCT116 and RKO, TZP exerted a concentration‐dependent inhibition of CRC cell migration and tube formation (Figure S2, Supporting Information). To assess the specificity of its antitumoral effects, a cell viability assay was used to examine its effects on four types of other cancer cell lines. TZP did not significantly inhibit the proliferation of glioma cells (U87MG), human gastric cancer cells (HGC27 and MKN45), and pancreas‐02 cells (Pan02) (Figure S3, Supporting Information), indicating that CRC cells were most sensitive to TZP.

**Figure 1:**
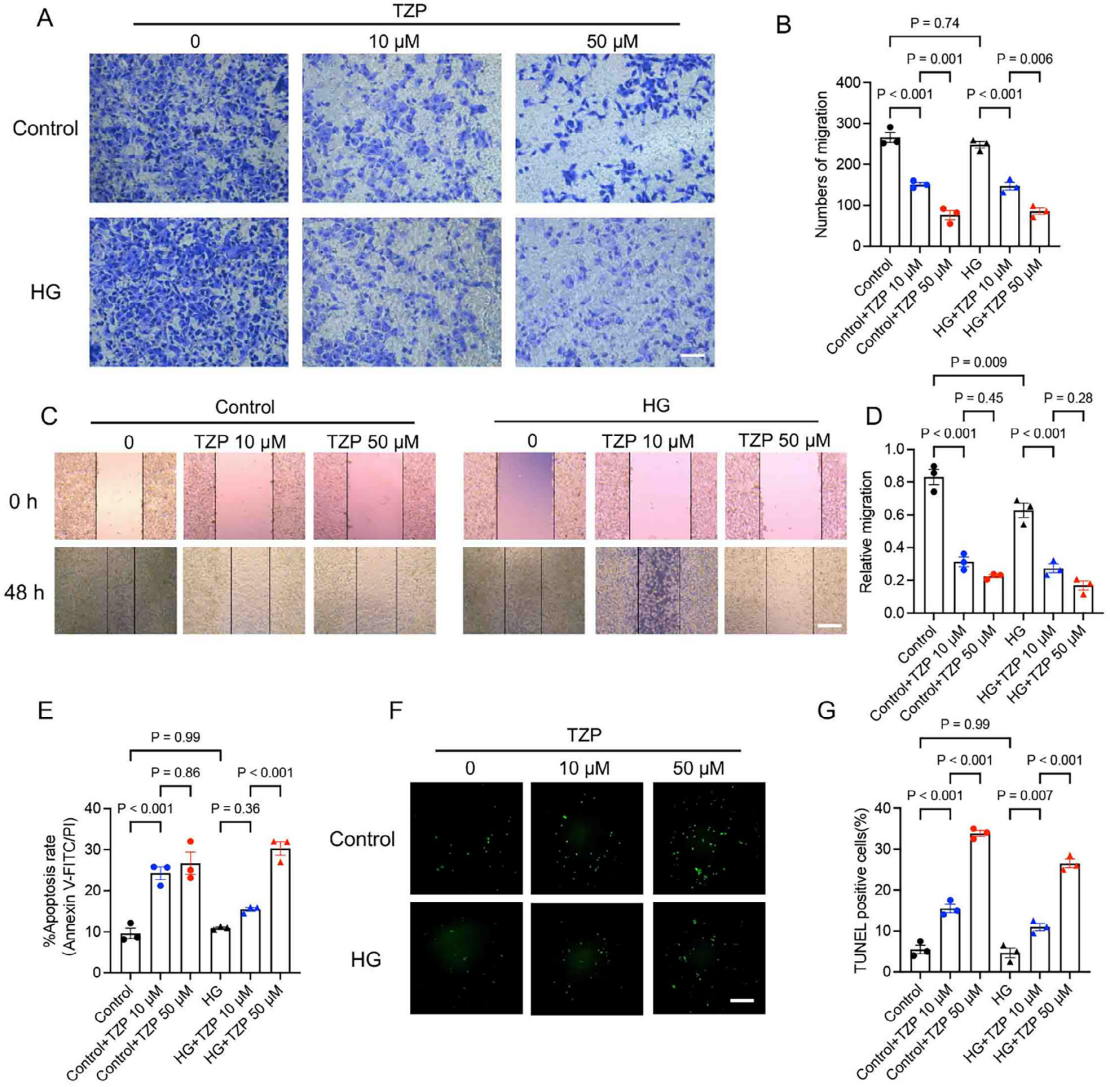
TZP inhibited CRC cell growth and migration under hyperglycemic and non‐hyperglycemic conditions in vitro. MC38 cells with normal glucose (2 mM) (Control group) or high glucose (33 mM) (HG group) were treated with TZP in 10 or 50 µM for 48 h. Cancer cell migration (invasiveness) and apoptosis were then measured. (A‐B) Migration/invasion was evaluated by the transwell assay (*n* = 3) (Scale bar: 200 µm). (C‐D) Migration was assessed by the wound healing assay (*n* = 3) (Scale bar: 200 µm). (E) Cells were stained with annexin V‐FITC and PI for flow cytometric analysis (*n* = 3). Percent apoptosis was quantified. (F‐G) TUNEL‐stained cells indicated apoptosis via green fluorescence. TUNEL‐positive cells were counted in a total of 200 cells in randomly selected fields (*n* = 3) (Scale bar: 100 µm). Data were shown as mean ± SEM. One‐way ANOVA was performed, and P values were adjusted with the Tukey multiple comparisons test (B, D, E, G).

As TZP is clinically used as an antidiabetic medication, we questioned whether its antitumor efficacy is related to glucose concentration. An in vitro model of MC38 under high glucose (HG) stress was established in the trans‐well system. TZP inhibited migration of the cancer cells across the permeable membrane in a dose‐dependent manner, and HG stimulation did not have an apparent effect (**Figure** **1**A,B). In the wound healing assay, cell migration in TZP‐treated groups decreased at both concentrations against the control cells but was more pronounced at 50 µM (Figure 1C,D). Flow cytometry and TUNEL assays revealed that TZP significantly increased the apoptotic ratio of MC38 cells, and this effect was independent of HG stimulation (Figure 1E–H).

### 2.2 Tirzepatide Delayed CRC Development Both in Diabetic Mice and Nondiabetic Mice

**Figure 2:**
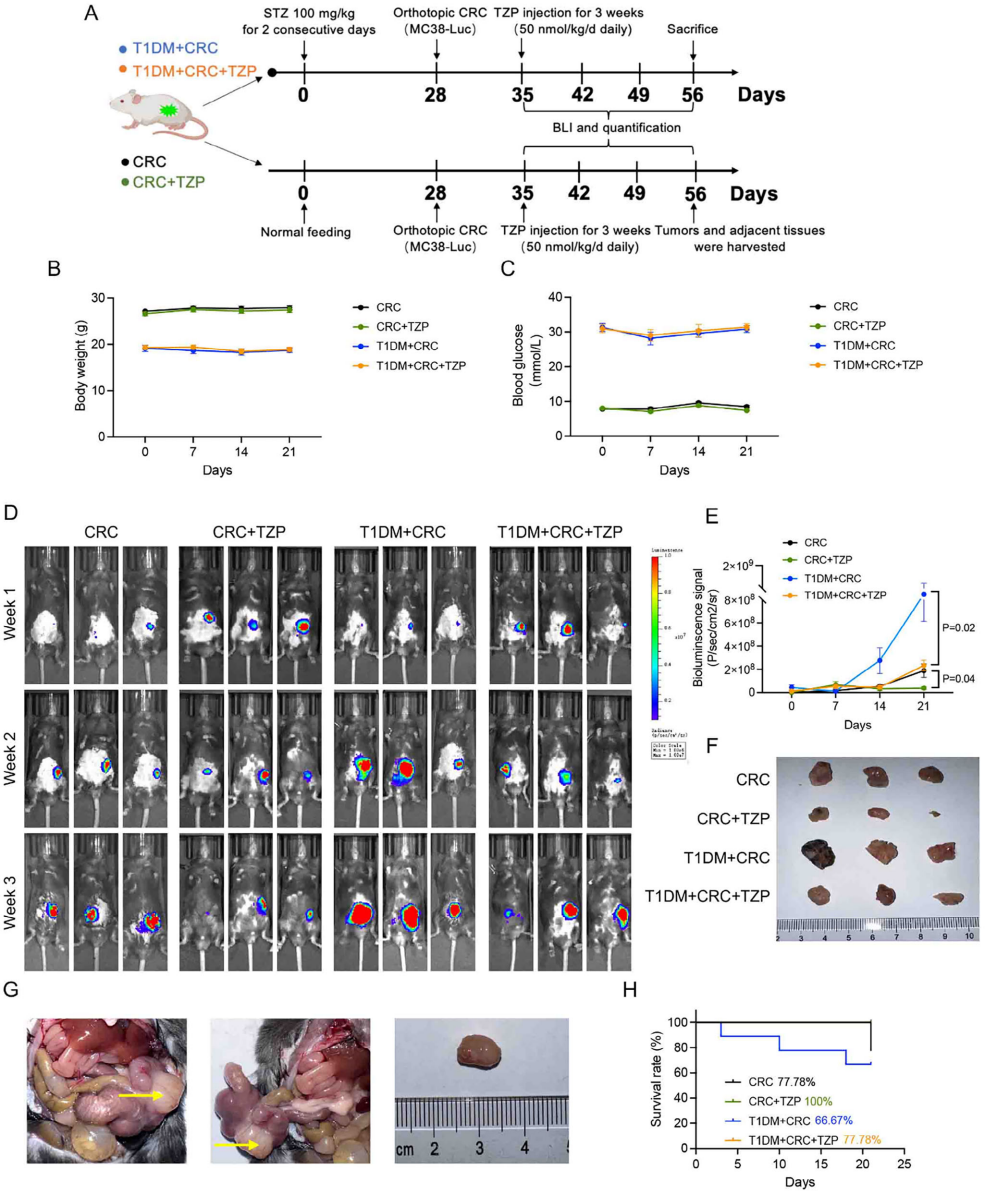
TZP inhibited CRC progression in T1DM mice independent of glycemic control. The effects of TZP on CRC were investigated in type 1 diabetic mellitus (T1DM) mice or control mice in the orthotopic model (*n* = 6–9). A) Schematic diagram of the animal model construction process. Mice were treated with STZ (100 mg kg^−1^) or without STZ (Control group) for 2 consecutive days. After stabilization of the blood glucose, mice were inoculated with MC38 cells to construct the orthotopic model. TZP was administered via intraperitoneal injection for a duration of 3 weeks (50 nmol kg^−1^ day^−1^ daily). B) Body weight and C) nonfasting blood glucose levels were measured after STZ injection. D) Bioluminescence radiant efficiency versus time. Increased total signal intensity over time suggested the progression of cancer. E) Longitudinal bioluminescence imaging of mouse MC38‐Luc derived tumors in different groups of mice. F, G) Representative examples of primary colon tumors at necropsy and size of the tumors 3 weeks after cell inoculation in the colon. Arrows indicated the tumors. H) Survival rate of tumor‐bearing mice in different groups. Data were shown as mean ± SEM. Statistical significance was determined by one‐way repeated measurement ANOVA with Tukey multiple comparisons test (E).

**Figure 3:**
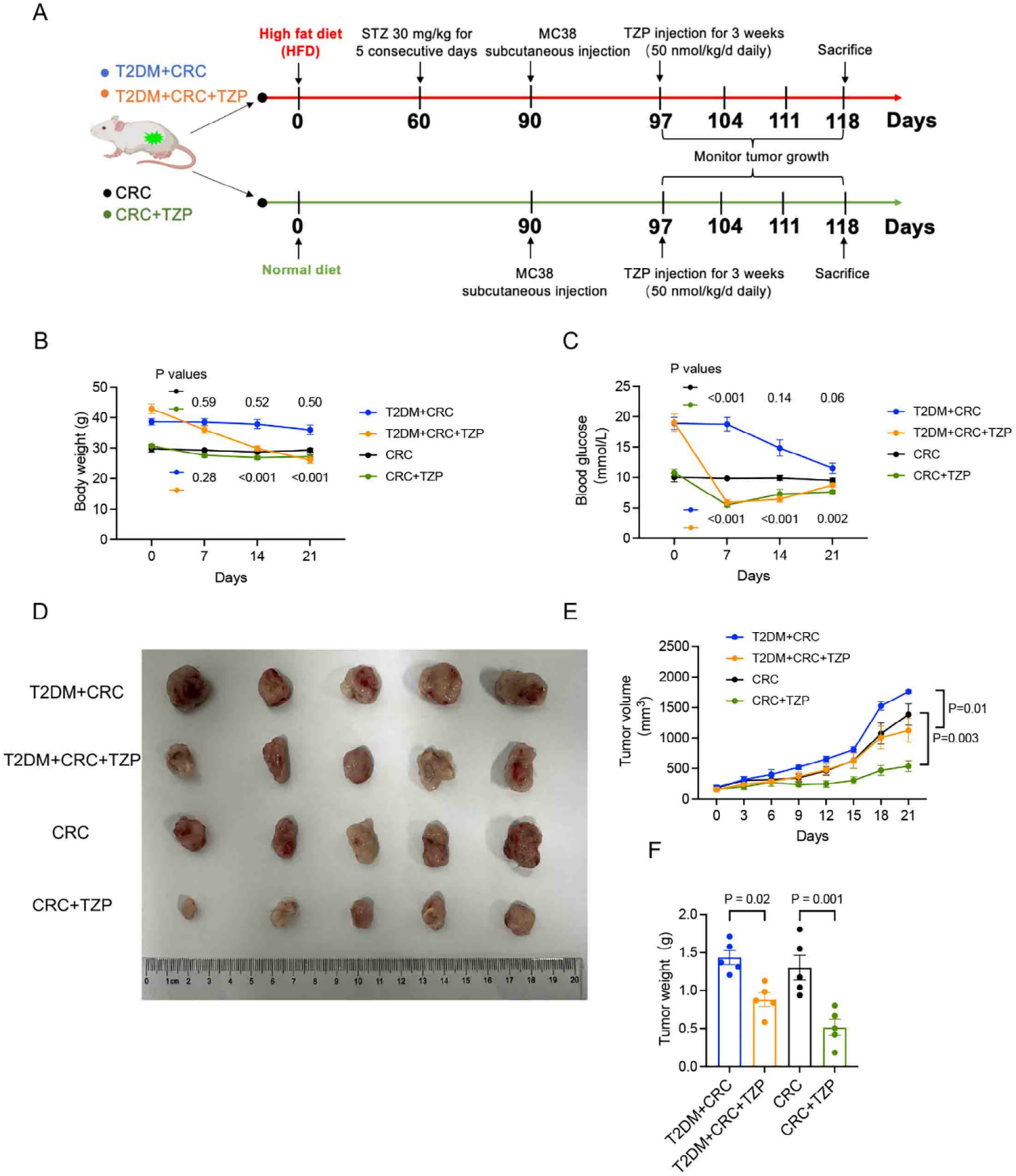
TZP delayed CRC proliferation both in T2DM mice and non‐diabetic mice. The effect of TZP on CRC was investigated in type 2 diabetic mellitus (T2DM) mice or control mice in the subcutaneous model. A) Schematic diagram of the animal model construction process. T2DM group mice were fed with a high‐fat diet for up to 8 weeks and control mice were fed with a normal diet. T2DM group mice then were injected with STZ (30 mg kg^−1^) for 5 consecutive days. After stabilization of the blood glucose, mice were inoculated with MC38 cells to construct the subcutaneous animal model. B) Body weight and C) fasting blood glucose level were measured after STZ injection. (*n* = 5–8) D) Images of isolated tumors from the subcutaneous tumor mice. E, F) Tumor weight and volume were observed and recorded. (*n* = 5) Data were shown as mean ± SEM. Statistical significance was determined by one‐way repeated measures ANOVA with Tukey multiple comparisons test (B, C, E, F).

To explore if the anti‐tumoral effect of TZP would occur in vivo, orthotopic and subcutaneous animal models were constructed in normal mice and STZ‐induced T1DM and T2DM mice (**Figures** **2**A and **3**A). TZP was administered via intraperitoneal injection every day (50 nmol/kg/day) for 3 weeks according to the half‐life of TZP in mice (Figure S4, Supporting Information). TZP did not affect body weight and blood glucose in T1DM and control mice (Figure 2B,C). Treatment with TZP inhibited tumor growth of CRC, either diabetic or non‐diabetic, as revealed by in vivo imaging and tumor volume measurement (Figure 2D–G), indicating that the inhibitory effect of TZP on CRC in vivo is independent of blood glucose regulation. And TZP increased the survival of T1DM mice with CRC (MC38‐Luc) (Figure 2H). Next, we constructed subcutaneous models using murine colon cancer cells (MC38) in control mice and T2DM mice. We found that treatment with TZP significantly reduced blood glucose levels, body weight (Figure 3B,C), decreased fatty acid levels, and some degree of reduction of caloric intake during 7–14 days of TZP injection with T2DM mice (Figure S5A, Supporting Information). A slight reduction of plasma insulin levels was possibly due to the rescue in insulin sensitivity (Figure S5B,C, Supporting Information). The tumor volume in diabetic mice was the largest while TZP treatment significantly reduced CRC volume and weight in T2DM mice (Figure 3D–F). It might be that tumor progression and drug response require extended time periods, and short‐term studies may not capture significant weight changes in control mice. Besides, the presence of cancer might compromise body weight.

### 2.3 TZP Reduced Glycolytic Metabolism in the Colon Cancer Tissues

**Figure 4:**
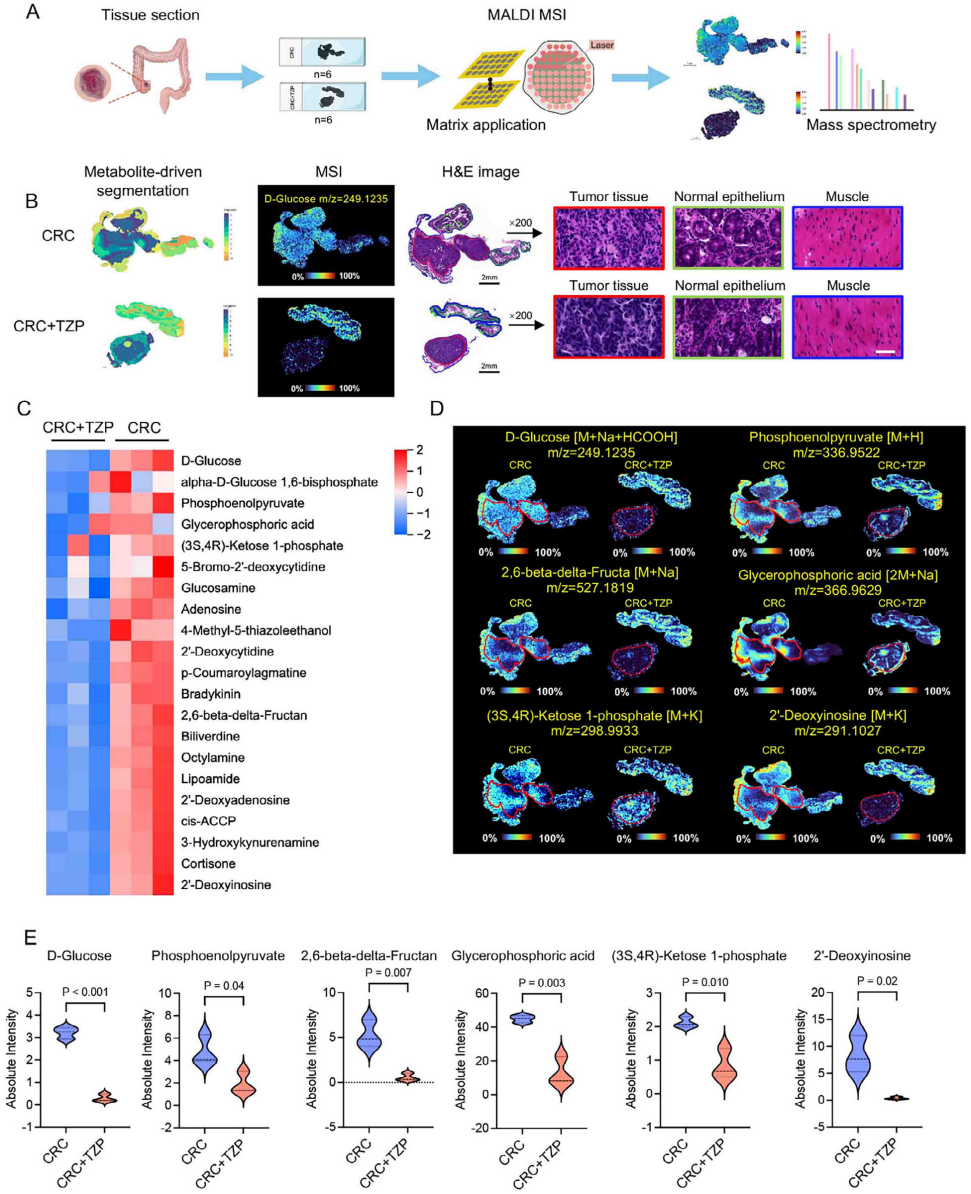
Spatial metabolomics profiled shows that TZP reduced glycolysis in the colon cancer tissues. A) Illustration of colonic and peritumoral tissues used for spatial metabolomic datasets. B) Examples of the microscopy‐mass spectrometry imaging (MSI) overlay. MSI images of D‐glucose (m/z = 249.1235) and H&E images of CRC tissue sections. Scale bar: 50 µm C) Heatmap of glycolysis‐related metabolites across all experimental animals of both groups. Red represented higher levels and blue, lower levels. D) Region‐specific MS images of CRC tissue sections. E) Violin plotting to show data distribution and its probability density; the thin black line extending from it, the 95% confidence interval; the black horizontal line in the middle, the median; and the outer shape, the distribution density of the data (*n* = 3). Statistical significance was determined by unpaired t‐test and *p* values were adjusted with the t unpaired test comparisons test.

Accumulating evidence demonstrates the important roles of glucose metabolism in determining cancer cell fate.^[^ ^15^, ^17^ ^]^ We used spatial metabolomics to further characterize whether TZP exerted its anti‐colon cancer effects by influencing metabolism. We used in situ Matrix‐Assisted Laser Desorption/Ionization Mass Spectrometry (MALDI‐MS) imaging of all CRC specimens from the orthotopic mice model (Figure 2A) to visualize and quantify metabolites to identify the enriched metabolomic profiles in tumor regions (**Figure** **4**A). Postoperative CRC tissue sections were divided into three histologic types based on the cell type and components: cancer tissue, epithelial tissue, and muscular tissue (Figure 4B). Multivariate analysis and volcanic map of metabolites showed that TZP treatment reduced the vast majority of the metabolite differences in the cancer tissue, including downregulation of 541 metabolites and upregulation of only 4 metabolites (Figure S6A,B, Supporting Information). The KEGG pathway analysis indicated that TZP treatment led to the activation of the metabolism‐related pathways and those related to cancer, cell cycle, etc. (Figure S6C,D, Supporting Information). A multitude of region‐specific glucose metabolites were screened and visualized, and the downregulation of products in the CRC tissues was apparent by TZP treatment (Figure 4C). Some key glycolytic metabolites in the tumor tissues were significantly downregulated after TZP treatment, such as D‐glucose, phosphoenolpyruvate, 2,6‐beta‐delta‐fructan, glycerophosphoric acid, (3S,4R) ‐ketose 1‐phosphate and 2′‐deoxyinosine (Figure 4D,E). These findings indicated that TZP robustly reduced glycolytic metabolism in the CRC tissues.

### 2.4 TZP Deprived the CRC Tissues of Glucose Metabolism by Inhibiting PFKFB3 and PFK‐1

**Figure 5:**
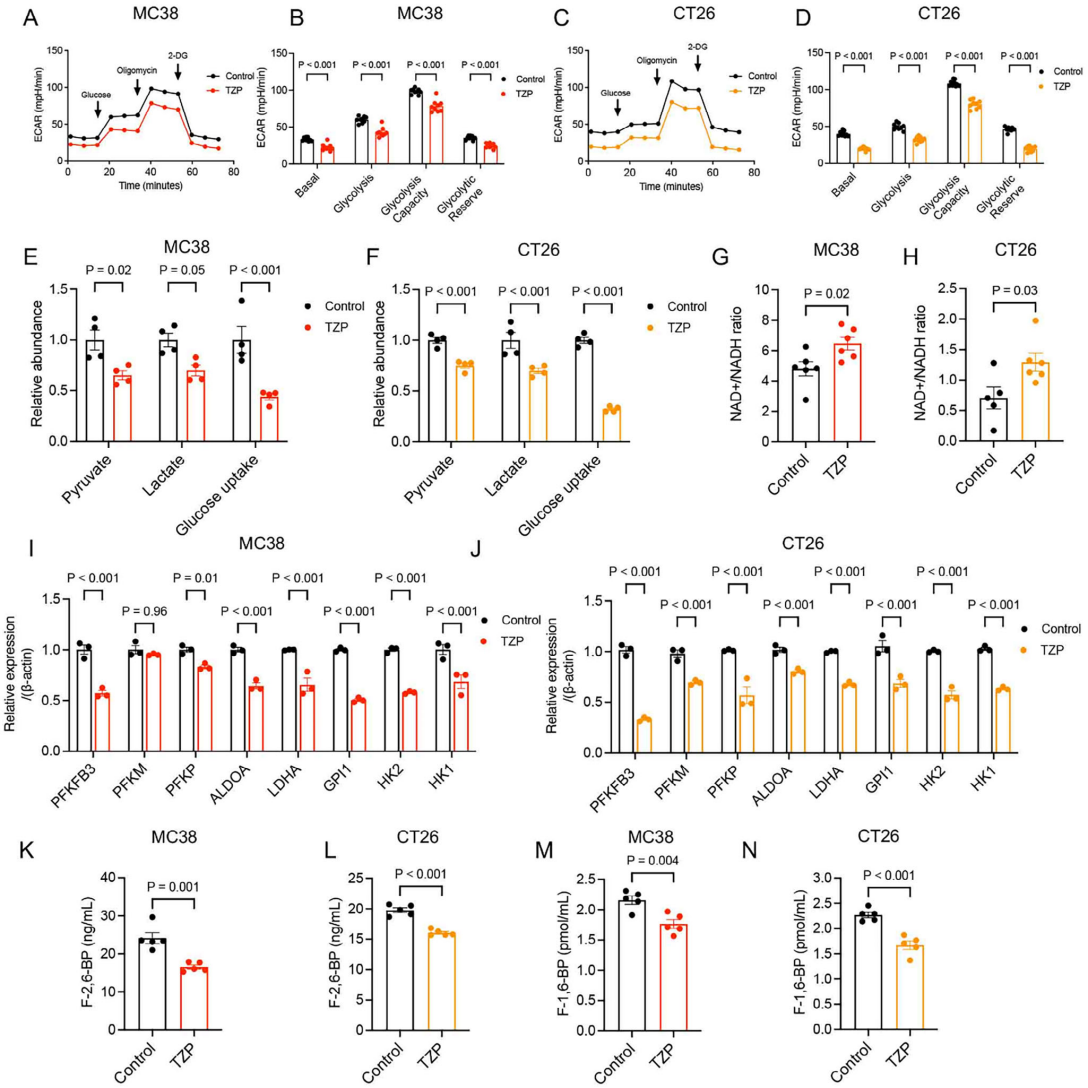
TZP attenuated glucose flux and transcription of glycolysis‐related genes. MC38 and CT26 cells were divided into 2 groups (Control vs TZP). (A‐D) Glycolytic flux (extracellular acidification rate [ECAR] using Seahorse XF) was measured separately (*n* = 9 per time point). Representative basal respiration, glycolysis, glycolysis capacity, and glycolytic reserve were shown. Quantification of ECAR was calculated (*n* = 9). (E‐F) Quantification of pyruvate, lactate, and glucose uptake in MC38 and CT26 cells. (*n* = 4) (G‐H) Analysis of NAD+/NADH ratios in MC38 and CT26 cells (*n* = 6); (I‐J) mRNA changes were quantified by qPCR of selected glycolytic genes (*n* = 3). (K‐L) Concentration of fructose‐2,6‐bisphosphate (F‐2,6‐BP) in MC38 and CT26 cells (*n* = 5). (M‐N) Concentration of fructose‐1,6‐bisphosphate (F‐1,6‐BP) in MC38 and CT26 cells (*n* = 5). Data were shown as mean ± SEM. Statistical significance was determined by unpaired *t*‐test (G, H, K‐N). Statistical significance was determined by two‐way repeated measurement ANOVA with Tukey multiple comparisons test (B, D, E, F, I, J).

We performed further in vitro experiments using two murine colon cancer cell lines (MC38 and CT26) to investigate changes in the glycolytic metabolism mediated by TZP. Seahorse assay confirmed that TZP attenuated glycolytic flux (extracellular acidification rate [ECAR]) in MC38 and CT26 cancer cells (**Figure** **5**A–D). Pyruvate and lactate production, and glucose uptake were significantly reduced in the presence of TZP in multiple CRC cell lines (Figure 5E,F; Figure S7, Supporting Information). Decreased glycolysis consumes less NAD^+^ to generate NADH. As expected, diminished aerobic glycolysis induced by TZP led to an increase NAD^+^/NADH ratio in the cytoplasm (Figure 5G,H). The majority of the genes involved in the aerobic glycolytic pathway were significantly inhibited by TZP (Figure 5I,J; Table S1, Supporting Information). TZP affected the enzyme activity of PFKFB3 and PFK‐1 which are engaged in the rate‐limiting step in glucose metabolism (Figure 5K–N). We also investigated the inhibitory effects of TZP on glycolysis levels and on glucose transporters GLUT1, GLUT3, GLUT4, and GLUT12 in HCT116. The results indicated that TZP reduced glycolysis and downregulated the expression of glucose transporters at mRNA and protein levels in HCT116 (Figure S8, Supporting Information). Interestingly, the stable isotope tracing found that TZP significantly decreased the enrichment of [U‐^13^C_6_] glucose‐labeled M+3 lactate and M+2 citrate, aconitate, isocitrate, succinate, and malate, indicating the blockage of tricarboxylic acid (TCA) cycle (Figure S9, Supporting Information). These findings suggested that TZP inhibited both glycolysis and the TCA cycle, thereby impairing the energy production in the CRC cells.

### 2.5 TZP Promoted HIF‑1α Ubiquitination and Degradation to Interrupt Glycolysis in Colon Cancer

**Figure 6:**
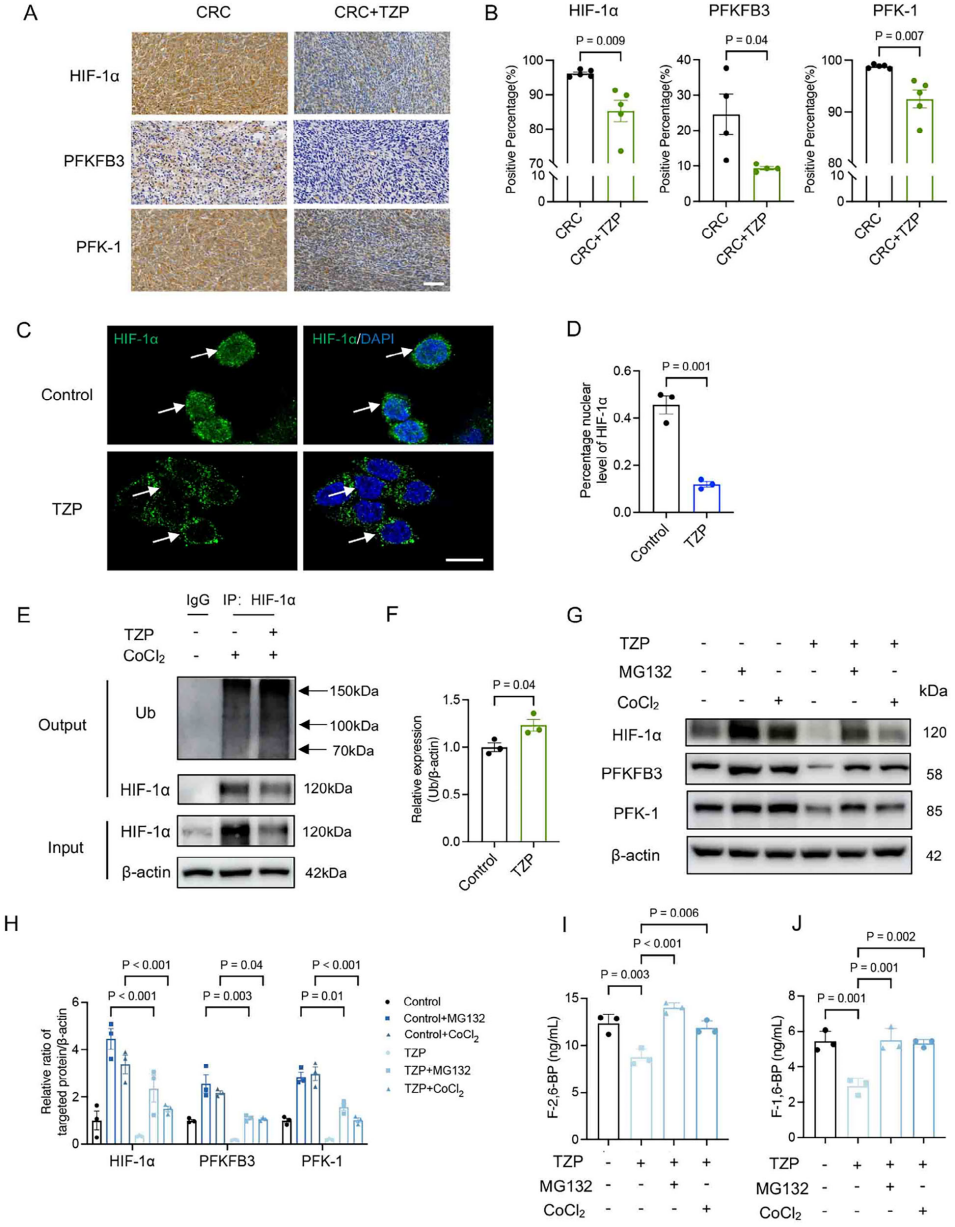
TZP promoted HIF‑1α ubiquitination and degradation to inhibit PFKFB3‐PFK‐1 mediated glycolysis. A, B) Representative immunohistochemical images of subcutaneous tumor tissues showing the expressions of HIF‐1α, PFKFB3, and PFK‐1. Scale bar: 500 µm. (*n* = 5). C, D) Immunofluorescence demonstrated the nuclear levels of HIF‐1α (*n* = 3). Scale bar: 50 µm. E, F) MC38 cells were cultured with CoCl_2_ (500 µM) for 6 h to induce the hypoxia or treated with MG132 (5 µM), a proteasome inhibitor, for 6 h and then incubated with or without TZP (50 µM). Co‐Immunoprecipitation assay was used to detect the ubiquitination level of HIF‐1α. Ratios of Ub‐HIF‐1α to β‐actin (*n* = 3). G, H) Expressions of HIF‐1α, PFKFB3, and PFK‐1 were assayed by western blotting. Ratios of HIF‐1α, PFKFB3 and PFK‐1 to β‐actin (*n* = 3). Data were shown as mean ± SEM. Statistical significance was determined by unpaired t‐test (B, D, F). Statistical significance was determined by one‐way repeated measurement ANOVA with Tukey multiple comparisons test (H, I, J).

We hypothesized whether TZP modulated glycolysis by targeting HIF‐1α, as it is considered a key regulator in cancer cell glucose metabolism^[^ ^18^ ^]^ and controls the transcription of glycolysis‐related genes.^[^ ^19^ ^]^ Immunohistochemistry results indicated that TZP reduced the expression of HIF‐1α, PFKFB3, and PFK‐1 in CRC tissues in vivo (**Figure** **6**A,B). Immunofluorescence analysis revealed that TZP treatment effectively blocked the translocation of HIF‐1α into the nuclei of the HCT116 cells as compared with the control cells (Figure 6C,D). We then investigated whether the reduced HIF‐1α expression by TZP was due to increased ubiquitination and enhanced protein degradation. To examine the effect of TZP on HIF‐1α stability, cobalt chloride (CoCl_2_) was used as HIF‐1α inducer and carbobenzoxyl‐L‐leucyl‐L‐leucyl‐L‐leucine (MG132), a proteasome inhibitor. Western blotting demonstrated that CoCl_2_‐induced upregulation of HIF‐1α and its ubiquitination was inhibited by TZP (Figure 6E,F). Wound healing and tube formation assays also confirmed that TZP regulated CRC function by affecting HIF‐1α stability (Figure S10, Supporting Information). TZP treatment could markedly reduce HIF‐1α as well as the key glycolytic molecules PFKFB3 and PFK‐1 in the HCT116 cells with or without (CoCl_2_) induction (Figure 6G,H). Meanwhile, TZP inhibited the enzymatic activity of PFKFB3 and PFK‐1, and this effect was counteracted by the stabilization of HIF‐1α (Figure 6I,J). Treating CRC cells with chemical inhibitors of PFKFB3 (PFK‐158, 10 µM) and HIF‐1α (LW6, 50 µM) or lentivirus to specifically knockdown PFK‐1, the results indicated that inhibition of the expression or transcription of these three key molecules significantly suppressed CRC cell migration and proliferation (Figures S11 and S12, Supporting Information). These results provided strong evidence that TZP played its anti‐CRC effect by targeting HIF‐1α degradation leading to reduced glycolysis with the inhibition of PFKFB3 and PFK‐1.

### 2.6 TZP Attenuated Colorectal Cancer Development and Downregulated HIF‐1α, PFKFB3, and PFK‐1 in PDX Models

**Figure 7:**
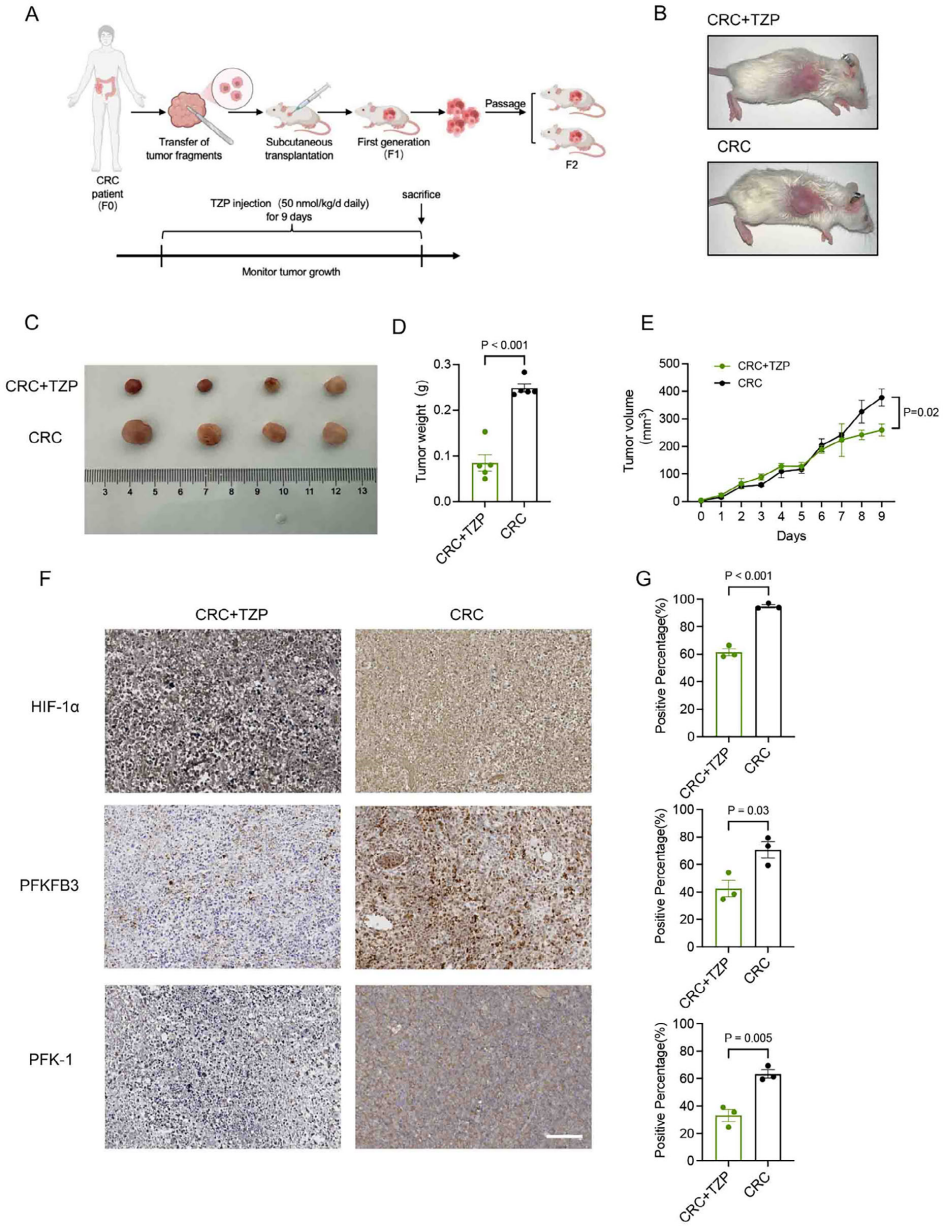
TZP attenuated colorectal cancer development and downregulated HIF‐1α, PFKFB3, and PFK‐1 in the patient‐derived xenograft model. A) Experimental workflow of CRC‐PDX (colorectal cancer and patient‐derived xenograft sample processing in mice treated with TZP (*n* = 4–6) (B) Images of PDX mice models. (C) Images of isolated tumors from the subcutaneous tumor mice using PDX fragments (≈25 mm^3^). (D‐E) Tumor weight and volume were observed and recorded (*n* = 5). (F‐G) Representative immunohistochemical images of PDX tumor tissues showing the expressions of HIF‐1α, PFKFB3, and PFK‐1(*n* = 3). Scale bar: 100 µm. Data were shown as mean ± SEM. Statistical significance was determined by unpaired t‐test (D, E, G).

As low‐passage patient‐derived xenograft (PDX) tumor models better reflect human tumor cell heterogeneity than do conventional cell lines and xenografts and are useful for assessing in vivo response to therapeutic agents,^[^ ^20^ ^]^ we surgically resected human CRC tissues and subcutaneous transplantation to NOD/ShiLtJGpt‐*Prkdc*em^26Cd52^ *Il2rg* ^em26Cd2^2/Gpt (NCG mice) to conduct CRC‐PDX model to evaluate the therapeutic efficacy of TZP (**Figure** **7**A). Due to the different genetic backgrounds of NCG and varying sensitivity to TZP, we adjusted the TZP injection duration for 9 days accordingly. The results showed that TZP injection significantly delayed the tumor growth as compared with the control group, consistently causing a significant reduction in the tumor volume and weight (Figure 7B–E). Immunohistochemistry was performed to detect the expression of HIF‐1α, PFKFB3, and PFK‐1 in the tumor tissues of both groups. Consistent with previous findings in the cell‐line derived cancer model (Figure 6A,B), TZP significantly reduced the expression of the target proteins in tumor tissues, confirming that TZP inhibited the HIF‐1α‐mediated glycolytic pathway in human tumor cells (Figure 7F,G).

## 3 Discussion

Obesity and diabetes are well‐known risk factors for various cancers.^[^ ^21^ ^]^ TZP, designed to act on both the GIP and GLP‐1 receptors, has recently emerged as a promising injectable drug for T2DM treatment and received FDA approval following successful clinical trials (SURPASS1/2/3/4 and 5, NCT03954834, NCT03987919, NCT03882970, NCT03730662, and NCT04039503).^[^ ^5^, ^22^ ^]^ Beyond its strengths on T2DM observed in clinical practice, whether TZP, when used as an anti‐diabetic drug in diabetic patients, could benefit cancer therapeutics or increase the risk of cancer progression remains unexplored. Here, we reported that TZP attenuated colon cancer proliferation and tumor growth in multiple CRC cells and murine models and exhibited the potential as a monotherapy against colorectal cancers under hyperglycemic and non‐hyperglycemic conditions. Mechanically, we found that TZP exerted its anti‐CRC effect by inhibiting glucose influx and targeting HIF‐1α for degradation with reduced expression of its downstream glycolytic gene *PFKFB3* and *PFK‐1*, leading to reduced glycolysis and TCA in the cancer tissues.

Because of the recognized link between high blood glucose levels/diabetes and obesity to the incidence of cancer and its progression, we first investigated whether the effects of TZP against colon cancer cells differ under normal and HG conditions. We found that TZP exhibited a concentration‐dependent inhibitory and pro‐apoptotic effect in MC38, HCT116, and RKO cells (Figures S1 and S2, Supporting Information). To exclude the potential indirect antitumor effects of TZP mediated through blood glucose modulation, we established a T1DM mouse model implanted with colon cancer to evaluate its direct anti‐tumor efficacy. The results demonstrated that under comparable glycemic conditions, TZP significantly inhibited tumor proliferation in both T1DM mice and normoglycemic control mice (Figure 2). Additionally, we found that TZP affected the growth of mouse orthotopic or ectopic colon cancer as well as the PDX model, suggesting its inhibitory effects on diverse colon cancer models in vivo. Recent studies have highlighted the potential to further enhance the clinical benefits of monotherapies by combining them with chemotherapeutic agents for synergistic action.^[^ ^23^ ^]^ In our study, TZP and oxaliplatin (OXA) combination strengthened the antitumor effect in MC38, and TZP combined with 5‐FU (5‐Fluorouracil) or SN‐38 (Irinotecan) also synergistically retarded CT26 growth (Figure S13, Supporting Information). These results suggested the potential for synergistic anti‐cancer effects when combined with chemotherapeutic agents.

In normal cells, such as pancreatic β‐cells and adipocytes, TZP has been confirmed as an effective insulin sensitizer to improve insulin sensitivity.^[^ ^24^ ^]^ However, tumor cells typically rely on a mechanism known as the “Warburg Effect,” where they preferentially generate energy through glycolysis even in the presence of sufficient oxygen.^[^ ^25^ ^]^ As altered energy metabolism is one of the “hallmarks of cancer”,^[^ ^15b^ ^]^ blocking the glycolytic pathway has been suggested as a therapeutic strategy to retard the proliferation of tumor cells.^[^ ^26^ ^]^ Therefore, we hypothesized that TZP might inhibit CRC tumor growth by altering the metabolic pathways. We found that TZP treatment changed the spatial distribution of glycolytic metabolites with reduced glycolytic metabolism in the colon cancer region, which was consistent with the anti‐tumor effect. In vitro studies using the MC38, CT26, and HCT116 cancer cell lines, TZP attenuated glycolytic flux (extracellular acidification rate [ECAR]), pyruvate and lactate production, and reduced glucose uptake. Importantly, TZP inhibited transcription and translation of glucose transporters (GLUT1, GLUT4, GLUT12) and decreased the levels of intermediates in the TCA cycle. Therefore, TZP might interfere with this pathway to reduce glucose uptake in tumor cells and downregulate PFKFB3 and PFK1, thereby limiting their growth (Figures 4, 5, 6). Considering the differences in metabolic pathways between normal and tumor cells, the mechanisms by which TZP affected energy metabolism in tumor cells could be distinct as well. We will continue to investigate the regulatory mechanisms and potential targets of TZP in tumor metabolism.

Hypoxia has been identified as an inherent impediment to cancer therapy due to its multiple contributions to chemoresistance, angiogenesis, and invasiveness properties.^[^ ^27^ ^]^ HIF‐1α is the key mediator of the transcriptional responses to hypoxia and plays an important role in cancer proliferation and poor clinical outcomes.^[^ ^28^ ^]^ The complexes of yes‐associated protein (YAP)/HIF‐1α could accelerate glycolysis under hypoxic conditions by binding to the glycolytic gene promoter.^[^ ^29^ ^]^ In this study, KEGG pathway analysis showed the enrichment of the HIF‐1 signaling pathway. We speculated from our findings that TZP might promote HIF‐1α ubiquitination and proteasomal degradation in CRC cells which, in turn, affected the transcription of glycolysis‐related genes. TZP inhibited the expression and enzymatic activity of PFKFB3 and PFK‐1, and such effects were not affected under hypoxic conditions induced by CoCl_2_ but counteracted by the stabilization of HIF‐1α. However, no inhibitory effect of TZP was observed in other cancer cell lines such as gastric cancer, liver cancer, and pancreatic cancer (Figure S3, Supporting Information). A crucial feature that sets CRC apart is the aberrant activation of the Wnt signaling pathway, a hallmark of this disease.^[^ ^25^ ^]^ A recent study revealed that Wnt3a‐induced rapid transactivation led to high expression of lactate dehydrogenase A (LDH‐A). LDH‐A bound to HIF‐1α and enhanced its stability by obstructing proteasome degradation, leading to increased transactivation of glycolytic genes.^[^ ^30^ ^]^ These findings, together with the heterogeneity of different cancer types, might account for the difference that colon cancer cells were more sensitive to TZP than the other types of cancer cells.

Since TZP is a dual receptor agonist of GLP‐1R and GIPR, there remain uncertainties regarding the effects of these two receptors on glucose uptake and intracellular glucose metabolism. Both GIPR and GLP‐1R belong to the Class B1 GPCR family that is involved in glucose regulation and metabolic control, and both promote insulin secretion, though their mechanisms are different.^[^ ^31^ ^]^ TZP, as the first GLP‐1R/GIPR dual agonist, significantly enhances weight loss and glycemic control through the simultaneous activation of both receptors.^[^ ^32^ ^]^ Its mechanism might be related to the complementary nature of the signaling pathways of both receptors.^[^ ^33^ ^]^ We assessed the expression of the dual receptors in the cell lines and in vivo tumor tissues involved in this study. We found that both GLP‐1R and GIPR were expressed in the CRC cells in vivo, and TZP treatment activated the expression of both receptors (Figure S14, Supporting Information). The TZP‐mediated reduction of CRC cell viability or apoptotic response that we observed here might result from its GLP‐1R‐mediated mechanism. The GLP‐1R agonist exendin‐4 (Ex‐4) induced mouse colon cancer CT‐26 cell apoptosis in vitro, augmented apoptosis induced by irinotecan, and increased tumor apoptosis in the mouse model.^[^ ^9^ ^]^ And it also induced endometrial cancer Ishikawa cell apoptosis.^[^ ^34^ ^]^ Increased apoptosis by GLP‐1R agonist liraglutide was also seen in CRC cells.^[^ ^35^ ^]^ In this study, TZP exerted inhibitory effects on proliferation in cancer cells (MC36, CT26, HCT116, and RKO), but did not affect the viability of mouse embryonic hepatocytes BNL CL.2 (Figures S1 and S2, Supporting Information).

**Figure 8:**
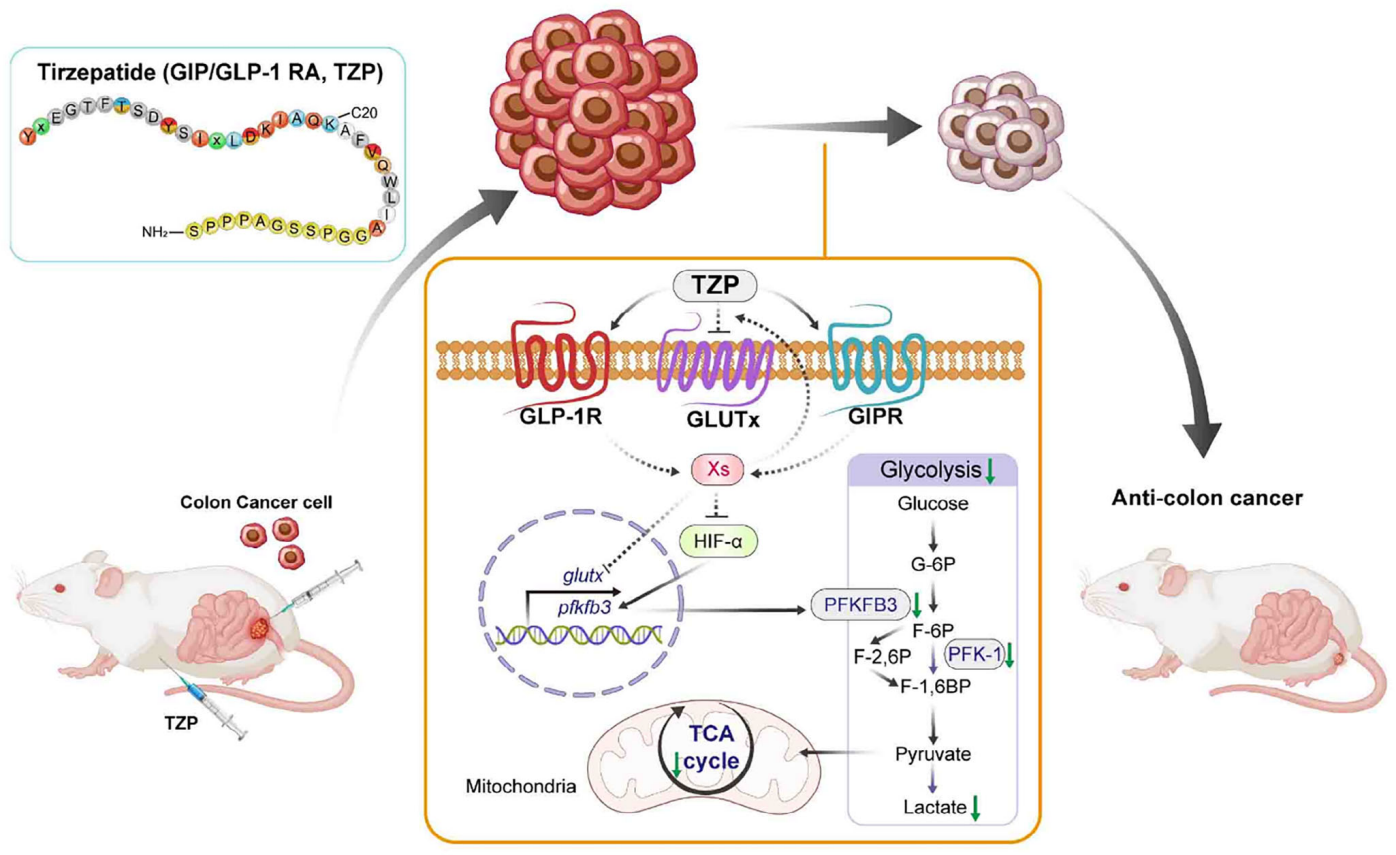
A proposed model of how TZP‐mediated dysregulation of glucose metabolism contributes to CRC progression. Titzepatide (TZP), a GIP/GLP‐1 receptor agonist, exerts its anti‐CRC effects by downregulating glucose metabolism (glycolysis and TCA cycle) through inhibiting GLUTx‐mediated glucose uptake via yet‐to‐be‐identified molecules (Xs) and by destabilizing HIF‐1α to inhibit PFKFB3‐PFK‐1.

This study explored the potential anti‐CRC effects and mechanisms of the dual receptor agonist TZP beyond its therapeutic role in type 2 diabetes. Through in vitro and in vivo experiments, we found that TZP significantly inhibited the proliferation and tumor growth in various CRC models, including multiple cell lines, orthotopic/subcutaneous tumors, and PDX models. This effect was independent of blood glucose levels. Mechanistic studies revealed that TZP suppressed the expression of key glycolytic enzymes, PFK‐1 and PFKFB3, by promoting the ubiquitination and degradation of HIF‐1α, significantly inhibiting the tumor tissues' glycolytic metabolic flux (evidenced by reduced ECAR and decreased pyruvate/lactate production), while also lowering the expression of glucose transporters (GLUT1/4/12) and the levels of TCA cycle intermediates. Notably, TZP demonstrated a synergistic anti‐tumor effect when combined with chemotherapy agents such as oxaliplatin and 5‐FU. Regarding the receptor mechanism, the study confirmed the co‐expression of GLP‐1R and GIPR in CRC tissues, and TZP treatment simultaneously activated both receptors, suggesting that its action may involve the coordinated regulation of both receptors signaling pathways. Although TZP, as the first GLP‐1R/GIPR dual agonist, has been approved by the FDA for the treatment of diabetes, its anti‐tumor mechanisms and the specific relationship with receptors activation to downstream molecules still require further exploration (**Figure** **8**). These findings provide a theoretical basis for the potential clinical application of TZP in CRC treatment and highlight the importance of metabolic regulation in colorectal cancer therapy.

## 4 Experimental Section

### Cell Culture

Murine colon carcinoma cell line (CT26 and MC38), human glioblastoma cell line (U87), human colon cancer cell line (HCT116 and RKO), human pancreatic adenocarcinoma cell line 02 (Pan02), human gastric carcinoma cell lines HGC27 and MKN45, and mouse embryonic hepatocytes line (BNL CL.2) were acquired from American Type Culture Collection (ATCC, Manassas, Virginia, USA). All cell lines were cultured with DMEM (Gibco; USA) supplemented with 10% (v/v) fetal bovine serum (FBS, Gibco) and antibiotic‐antimycotic (Gibco) at 37 °C in a 5% CO_2_ atmosphere except HCT116 cells were routinely maintained in McCoy's 5A Modified Medium (Gibco).

### Reagents and Chemicals

Tirzepatide (LY3298176) and cobalt chloride were purchased from Selleck (Houston, TX, USA). Proteasome inhibitor (MG132), PFKFB3 chemical inhibitor (PFK‐158), and molecule inhibitor of HIF‐1α (LW6) were purchased from MedChemExpress (NJ, USA). D‐glucose (NIST917C) was bought from Sigma (MO, USA). Lentivirus to specifically knock down PFK‐1 (Target sequence: GTG GCA CTG ATA TGA CCA TTG) was conducted by GenePharma (Shanghai, China)

### Cell Proliferation Assays

The proliferation of MC38, BNL, U87, Pan02, HGC27, and MKN45 cells was determined by a Cell Counting Kit‐8 assay (CCK8; Dojindo, Japan) as described.^[^ ^36^ ^]^ The cells of different lines were seeded in 96‐well plates and cultured for 12, 24, 48, 72, or 96 h (h) with different doses of TZP. Absorbance at 450 nm was examined using a microplate reader (Bio‐Rad, Berkeley, USA).

### Cell Migration Assays

Cell migration was assessed using Transwell permeable supports (Corning, Cat. No. 3421). Cells were suspended in serum‐free DMEM and seeded in the upper chamber, while DMEM containing 10% FBS was added to the lower chamber. After 48 h, migratory cells in the bottom chamber were stained with crystal violet, pictured, and counted by Adobe Photoshop 2024.

### Analysis of Wound Healing In Vitro

Cells were plated into wells of a 12‐well plate and cultured overnight until a confluent monolayer was formed, onto which a scratch was made with a 200 µL pipette tip. The effects of TZP (10 and 50 µM) on wound healing were measured at 0 and 48 h after wounding by imaging on the DMIL microscope system equipped with a DFC295 camera and controlled by LAS V4.0 software. (Leica, Germany). 1 µM of mitomycin (Selleck) was added to inhibit cell proliferation and eliminate its potential influence on the migration assay.

### Measurement of Apoptosis

Apoptosis was detected using an Annexin V‐FITC apoptosis detection kit (Vazyme), according to the manufacturer's instructions. After treatment, cells were trypsinized, washed twice with HBSS, resuspended in 1× binding buffer, and stained with Annexin V‐FITC and PI for 10 min at room temperature (RT) in the dark. At least 1 × 10^5^ cells were analyzed by flow cytometry, using side and forward scatter to identify viable cell populations.

For the TUNEL assay, the MC38 cells were treated with TZP in two different doses (10 and 50 µM) for 48 h. The cells were fixed with a 4% paraformaldehyde solution in PBS for 30 min at −20 °C, followed by permeabilization with 0.1% Triton X‐100 in 0.1% sodium citrate solution for 2 min on ice. Subsequent procedures were performed according to the manufacturer's protocol for the TUNEL BrightGreen apoptosis detection kit (Vazyme). Fluorescent images (magnification ×100) were acquired using a DMI8 fluorescence microscope (Leica, Germany).

### Animals

C57BL/6J male mice and PDX female NCG mice were brought from GemPharmatech (Nanjing, Jiangsu, China). The animals were maintained in a temperature‐controlled room (22 °C) on a 12‐h light‐dark cycle. The original PDX establishment followed the procedure approved by GemPharmatech's relevant committees and the original colon cancer samples were obtained from collaborating hospitals whose institutional committee approved the sample collection and obtained informed consent. Experimental setups and animal care were permitted by the Animal Policy and Welfare Committee of Zhejiang University (ethical approval code:2024‐NO.073).

### Diabetic Mice Model

C57BL/6J male mice were purchased from GemPharmatech (Jiangsu, China). For conducting T1DM model, mice at the age of 6 to 8 weeks were injected intraperitoneally of streptozotocin (STZ) (male mice: 100 mg kg^−1^ body weight, 1 dose a day for 2 days). For the T2DM model, mice were fed with a high‐fat diet (Research Diets, NJ, USA) for up to 8 weeks. On a caloric basis, the high‐fat diet consisted of 60% fat from lard, 20% carbohydrate, and 20% protein (total 5.24 kcal g^−1^), whereas the normal diet contained 11.1% fat, 67.4% carbohydrate, and 21.5% protein (total 3.41 kcal g^−1^). After 8 weeks of feeding, the control group was treated with sterile PBS, and the T2DM group was treated by intraperitoneal injection of STZ (male mice: 30 mg kg^−1^ body weight, 1 dose a day for 5 consecutive days). Blood glucose was measured once a week to validate diabetic hyperglycemia. Serum insulin was determined by immunoassay (Alpco Diagnostics, China), and serum‐free fatty acids were determined enzymatically (Nanjing Jiancheng Bioengineering Institute, China).

### Tumor Models and Treatments

For the orthotopic mouse model, 6‐ to 8‐week C57BL/6 male mice were anesthetized with 5% isoflurane. The hairs on the abdominal region were cleared before surgery, and the cecum was exposed. A 50 µL suspension of 5 × 10^5^ MC38 cells in Matrigel VR Basement Membrane (1:1 ratio) was carefully injected into the cecal wall from the serosal side under microscopic visualization. The cecum was then returned to the abdominal cavity, and the peritoneum, muscle, and skin closures were completed.^[^ ^37^ ^]^ TZP treatment started at day 35 by intraperitoneal injection daily for 21 days (50 nmol kg^−1^). The growth of tumors was monitored using the Xenogen IVIS 200 BLI system (Caliper Life Sciences, USA). Pictures were analyzed by Living Image 2.5 software.

For the subcutaneous animal model, C57BL/6J male mice were randomly assigned to groups of control and TZP (*n* =  10 per group), respectively. Equal numbers of corresponding cells (5  ×  10^5^ per mouse) were injected subcutaneously in the right flank to establish a CRC allograft model.^[^ ^38^ ^]^ When the tumors were palpable (7 days post‐injection), TZP treatment was initiated by intraperitoneal injection daily for 21 days using the same dose as described above. Tumor volume was measured every three days using a Vernier hand caliper. Tumor volume was calculated using the formula V = 0.52(length * width^2^). Mice were finally sacrificed at week 4 to harvest the tumor bulks.

CRC‐PDX (patient‐derived xenograft) establishment and treatment were similar to the above model. Surgically resected tissues were minced into pieces, ≈2‐mm in size, and injected into the subcutaneous area of the flanks of 6‐week NCG mice. Daily TZP injection lasted for 9 days. The tumor volumes of the mice were checked every day for 9 days.

### Spatially Resolved Metabolomics Analysis

Spatially resolved metabolomics profiling was conducted at MetWare Biological Science and Technology Co., Ltd. (Wuhan, China). Tissue samples were sectioned at a thickness of 12 µm using a Leica CM1950 cryostat (Leica Microsystems GmbH, Wetzlar, Germany) at −20 °C. The resulting tissue sections were then arranged on electrically conductive slides coated with indium tin oxide (ITO), and the slides were dried in a vacuum desiccator for 30 min.

For matrix coating, desiccated tissue sections mounted on ITO glass slides were sprayed using an HTX TM sprayer (Bruker Daltonics, Germany) with 15 mg mL^−1^ DHB (2,5‐dihydroxybenzoic acid), dissolved in 90% acetonitrile:10% water. The sprayer temperature was set to 60 °C, with a flow rate of 0.12 mL min^−1^, pressure of 5 psi. Thirty passes of the matrix were applied to slides with 5 s of drying time between each pass.

### Mass Spectrometry Imaging

MALDI timsTOF MSI experiments were performed on a prototype Bruker timsTOF flex MS system (Bruker Daltonics, Germany) equipped with a 10 kHz smartbeam 3D laser. Laser power was set to 80% and then fixed throughout the whole experiment. The mass spectra were acquired in positive mode. The mass spectra data were acquired over a mass range from m/z 50–1300 Da. The imaging spatial resolution was set to 50 µm for the tissues, and each spectrum consisted of 400 laser shots. MALDI mass spectra were normalized with the Root Mean Square, and the signal intensity in each image was shown as the normalized intensity. MS/MS fragmentations performed on the timsTOF flex MS system in the MS/MS mode were used for further detailed structural confirmation of the identified metabolites.

### Real‐Time Quantitative PCR

Total RNA of the tumors and control cells was extracted using TRIzol reagent (Invitrogen, USA), followed by cDNA synthesis using a reverse transcription kit (Yeasen, China) according to the manufacturer's instructions. Quantitative real‐time PCR was performed using a SYBR Green qPCR kit (Yeasen, China), and the primers were designed and synthesized by Tsingke Biotech Com (Shanghai, China). Primer sequences are listed in Table S1 (Supporting Information). Relative mRNA expression was normalized to β‐actin mRNA.

### Glycolysis Stress Test

As described in a previous publication,^[^ ^39^ ^]^ MC38, CT26, and HCT116 cells were seeded onto Seahorse XF96 culture cell plates at a concentration of 2 × 10^5^ per cell and incubated with 50 µM TZP at 37 °C overnight. On the second day, the treatment media were removed, and the cells were washed once with the assay medium (XF base medium supplemented with 2 mM glutamine, pH adjusted to 7.4). The culture medium was replaced with an assay medium containing the treatments, and the plates were incubated for 1 h in a non‐CO_2_ incubator at 37 °C. Following incubation, cellular analysis was performed using an XFe96 extracellular flux analyzer (Seahorse Bioscience). The following inhibitors and activators were applied: glucose (10 mM), oligomycin (1 µM), and 2‐DG (50 mM). Basel glycolysis, glycolytic capacity, and glycolytic reserve acidification were determined using the XF Wave 2.1 software.

### Measurement of Glucose Uptake, Lactate and Pyruvate Production

The effect of TZP on glucose uptake in each group of CRC cells was tested using the Glucose Uptake Fluorometric Assay Kit (E‐BC‐F041, Elabscience, China) according to the manufacturer's instructions. Briefly, cells were cultured and treated. Then, the cells were starved overnight in serum‐free cell medium and the next day, they were rinsed with KRPH solution plus 10 mmol L^−1^ 2‐DG and incubated for 30 min at 37 °C. The samples were mixed with reagent 4 working solutions and the fluorescent intensity was then measured at excitation/emission = 485/535 nm) and normalized to protein concentration measured using the Bio‐Rad protein assay (Bio‐Rad, USA).

Further, the glucose analog 2‐NBDG (MedChem Express, Monmouth Junction, NJ, USA) was also used to assess glucose uptake. Cells were treated with TZP (50 µM) for 48 h, after which the medium was replaced with PBS buffer containing 2‐NBDG (100 µM), and incubated at 37 °C for 30 min. The cells were then washed three times with PBS to eliminate residual extracellular 2‐NBDG. Subsequently, 2‐NBDG imaging was performed using a 488 nm laser and pictures were taken with an inverted fluorescence microscope (DMI8, Leica, Germany). Lactate and pyruvate production was measured using an enzymatic assay (Abcam, ab65331) and a pyruvate assay kit (Abcam, ab65342). All measurements were performed according to the manufacturer's instructions.

### Glucose Flux Measurements

The glycolytic flux was assessed by measuring the rate of glucose consumption and the ratio of ^13^C incorporated into lactate, determined using liquid chromatography‐mass spectrometry (LC‐MS). In brief, cells were cultured in a medium containing either D‐glucose‐^13^C_6_ or without it. After 12 h, the culture supernatants were collected, and cells were harvested using cold 80% methanol. Flux analysis was conducted on a TSQ Quantiva Triple Quadrupole mass spectrometer (Thermo Fisher Scientific, San Jose, CA) with positive/negative ion switching in multiple reaction monitoring (MRM) mode for data acquisition. Mobile phase A consisted of 2.376 mL tributylamine and 0.858 mL acetic acid in HPLC‐grade water, adjusted to a final volume of 1 liter. Mobile phase B was HPLC‐grade methanol. Separation of polar metabolites was achieved using a Synergy Hydro‐RP 100 A column at a column temperature of 35 °C. The mass isotopomer distributions were corrected for natural isotope abundances.

### ELISA Analysis

The cells were treated with cell lysis buffer for 10 min on the ice. The suspensions were then centrifuged at 120 00 g for 10 min at 4 °C, and the supernatant samples were used to measure the level of F‐2,6‐BP (mlbio, China) and F‐1,6‐BP using corresponding ELISA kits (mlbio, China) according to the manufacturer's instructions. Three replicate wells were used for each sample.^[^ ^40^ ^]^

### Immunohistochemistry (IHC)

To evaluate the levels of representative protein makers, immunohistochemical analyses were performed as described.^[^ ^41^ ^]^ In brief, tissues were fixed in 10% formalin and embedded in paraffin. Tumor tissues were fixed in 1 mL 4% paraformaldehyde overnight, then dehydrated in ethanol and embedded in paraffin. IHC was performed using the antibodies against HIF‐1α, PFK‐1, and PFKFB3. Reading of IHC slides and TMA was performed using Vectra 2 (Perkin Elmer) to quantify protein expression in the samples. For TMA, A Leica APERIO AT TURBO instrument was used for scanning and analysis. Quantification of IHC staining was performed blindly.

### Immunofluorescence Staining

Immunofluorescence analysis was performed briefly. The cells were seeded on glass coverslips, treated as indicated and fixed with 4% paraformaldehyde for 15 min at RT. Subsequently, the cells were washed three times with PBS and incubated with 1% BSA (Carl Roth) in PBS for 1 h at room temperature (RT). The coverslips were incubated with diluted primary HIF‐1α antibodies overnight at 4 °C, washed with PBS three times, and incubated with secondary antibodies for 1 h at RT. The cells were stained with 1× DAPI (Solarbio, China) for 5 min and washed three times with PBS. Imaging was performed with a STELLARIS 5 Cryo laser scanning confocal microscope (Leica, Germany).

### Western Blotting

Western blotting was conducted to investigate the expression and phosphorylation level of target proteins in the MC38, CT26, and HCT116 with different treatments. Protein lysates were extracted using radioimmunoprecipitation assay (RIPA) buffer (Beyotime, Haimen, China) and resolved by sodium dodecyl sulfate‐polyacrylamide gel electrophoresis (SDS‐PAGE). The proteins were transferred onto polyvinylidene fluoride (PVDF) membranes, blocked with QuickBlock Blocking Buffer (Beyotime), and incubated with primary antibodies at 4 °C overnight. After washing with TBST buffer, the membranes were incubated with horseradish peroxidase‐conjugated secondary antibodies for 1 h, followed by three washes with TBST. Immunoreactive bands were detected using SuperSignal West Pico chemiluminescent substrate (Thermo Scientific, Waltham, USA), and images were captured using a chemiluminescent imaging system (Amersham ImageQuant 800). Antibodies against β‐actin, HIF‐1α, PFKFB3, and GLUT1 were from Cell Signaling Technology (CST, Boston, USA) and Abcam (Cambridge, MA). Antibodies against PFK‐1 and GLUT3 were from Santa Cruz Biotechnology (Santa Cruz, California, USA), and those against GLUT4 and GLUT12, from Proteintech (Chicago, USA) and Affinity (Ohio, USA), respectively.

### In Vitro Ubiquitination Assays

The impact of TZP on the stability of HIF‐1α in colon cancer cells was examined by in vivo ubiquitination assays. Briefly, the different groups of cells were treated with CoCl_2_ (500 µM) under a normoxic condition for 8 h and the levels of HIF‐1α ubiquitination were determined by IP with anti‐HIF‐1α antibody, followed by western blotting using an anti‐Ub antibody (10201‐1‐AP, Proteintech, 1:1000).

### Statistics

Data are expressed as mean ± SEM from at least three independent experiments per condition. Significance testing was performed based on these data. Variance equality was assessed using the Brown‐Forsythe and Bartlett tests, while normality was evaluated with both normality and log‐normality functions in the software. If data met these criteria, comparisons among more than two groups were conducted using one‐way ANOVA with Tukey's multiple comparisons or two‐way ANOVA with Sidak or Tukey's multiple comparisons in GraphPad Prism (version 9.0). Two‐way repeated measures ANOVA was applied for repeated measurements from the same subjects under different conditions. Comparisons between the two groups were made using the unpaired *t*‐test, with a Family‐wise significance level of 0.05 and a 95% confidence interval for statistical significance.

## Conflict of Interest

The authors declare no conflict of interest.

## Supporting information



Supporting Information

## Data Availability

The data that support the findings of this study are available from the corresponding author upon reasonable request.
