# Supplementary material for: The Novel Dual GIP and GLP‐1 Receptor Agonist Tirzepatide Attenuates Colon Cancer Development by Regulating Glucose Metabolism
Source: Adv Sci (Weinh). 2025 Mar 24;12(19):2411980. doi: 10.1002/advs.202411980 (PMC12097124; doi:10.1002/advs.202411980)
Supplement: Supplementary file 1 — Supporting Information [file ADVS-12-2411980-s001.docx]

Supporting Information

Title: The novel dual GIP and GLP-1 receptor agonist tirzepatide attenuates colon cancer development by regulating glucose metabolism

Yikai Zhang^1^, Yi Xie^1^, Shenglong Xia^3^, Xinnuo Ge^1^, Jiaying Li^5^, Fang Liu^1^, Fan Jia^4^, Shengyao Wang^1^, Qiao Zhou^1^, Menghan Gao^1^, Weihuan Fang^2^, Chao Zheng^1*^

Y. Zhang, Y. Xie, X. Ge, F. Liu, S. Wang, Q. Zhou, M. Gao, C. Zheng

Department of Endocrinology, The Second Affiliated Hospital, School of Medicine, Zhejiang University

Hangzhou, 310009, P. R. China

E-mail: [chao_zheng@zju.edu.cn](mailto:chao_zheng@zju.edu.cn)

S. Xia

Department of Gastroenterology, The Second Affiliated Hospital, School of Medicine, Zhejiang University

Hangzhou 310009, P. R. China

J. Li

Center for Basic and Translational Research, The Second Affiliated Hospital, School of Medicine, Zhejiang University

Hangzhou 310009, P. R. China

F. Jia

MOE Key Laboratory of Macromolecule Synthesis and Functionalization of Ministry of Education, Department of Polymer Science and Engineering, Zhejiang University

Hangzhou 310009, P. R. China

W. Fang

Department of Veterinary Medicine, Zhejiang University

Hangzhou 310009, P. R. China


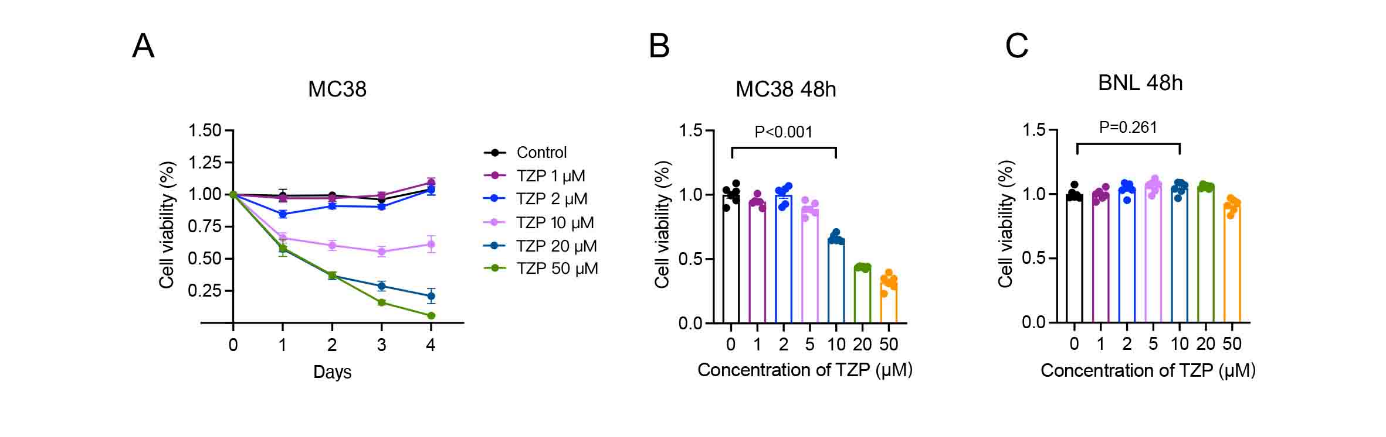


**Figure S1.** **The effect of TZP on viability of MC38 and BNL cells.**

MC38 and BNL cells were treated with TZP in different doses and cell viability was detected by CCK-8 assay. (A) MC38 cell viability during TZP treatment for 4 days. (B) MC38 cell viability at 48 h with TZP treatment. (C) BNL cell viability at 48 h with TZP treatment. Data were shown as mean±SEM. One-way ANOVA was performed, and P values were adjusted with the Tukey multiple comparisons test (B, C).


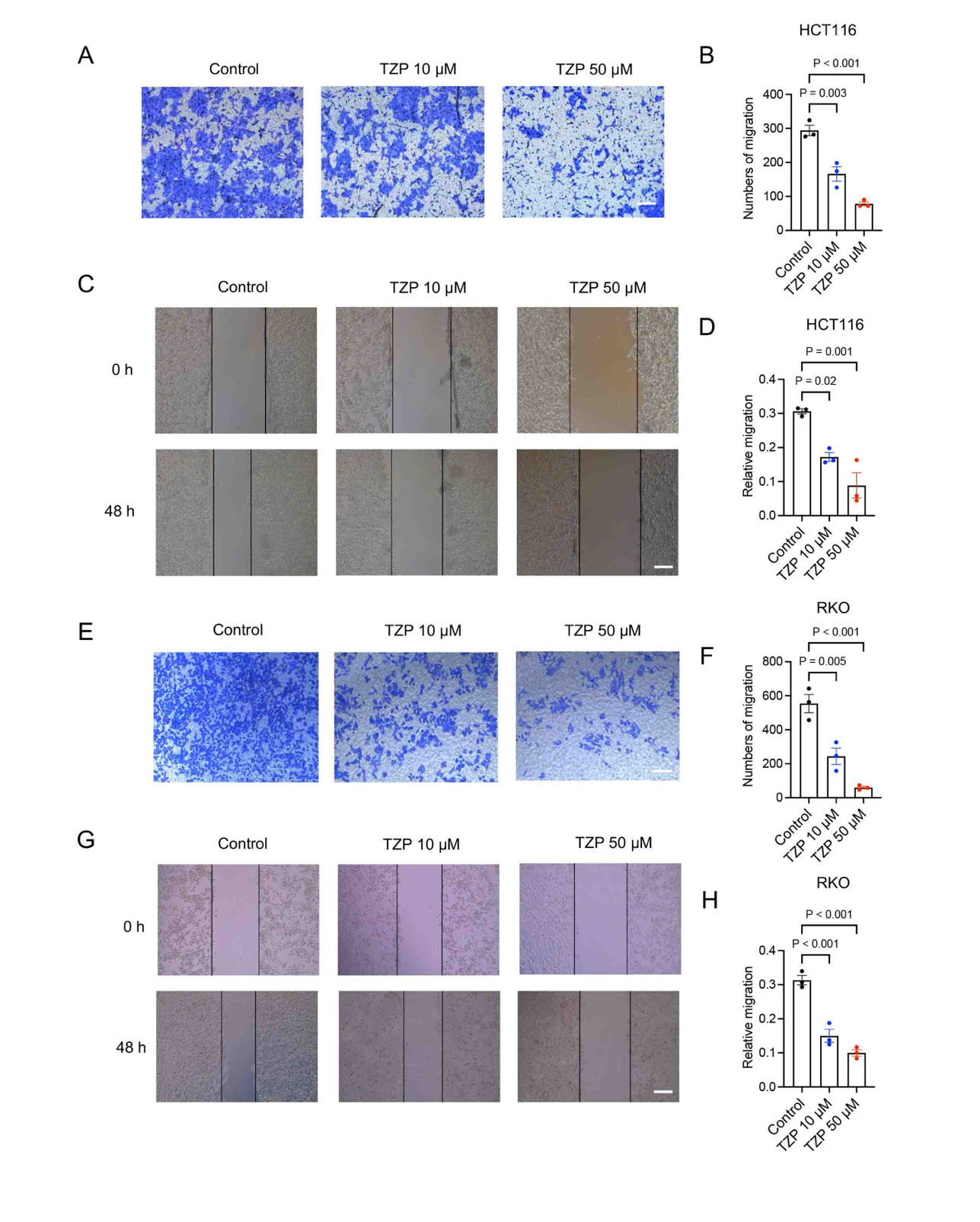


**Figure S2. The effects of TZP on cell migration and wound healing capacity evaluated in HCT116 and RKO colorectal cancer cells.**

HCT116 and RKO cells were treated with TZP in 10 or 50 µM for 48 h. (A-B, E-F) Migration/invasion was evaluated by the transwell assay (n=3) (Scale bar: 200 μm). (C-D, G-H) Migration was assessed by the wound healing assay (n=3) (Scale bar: 200 μm). Data were shown as mean±SEM. One-way ANOVA was performed, and P values were adjusted with the Tukey multiple comparisons test (B, D, F, H).


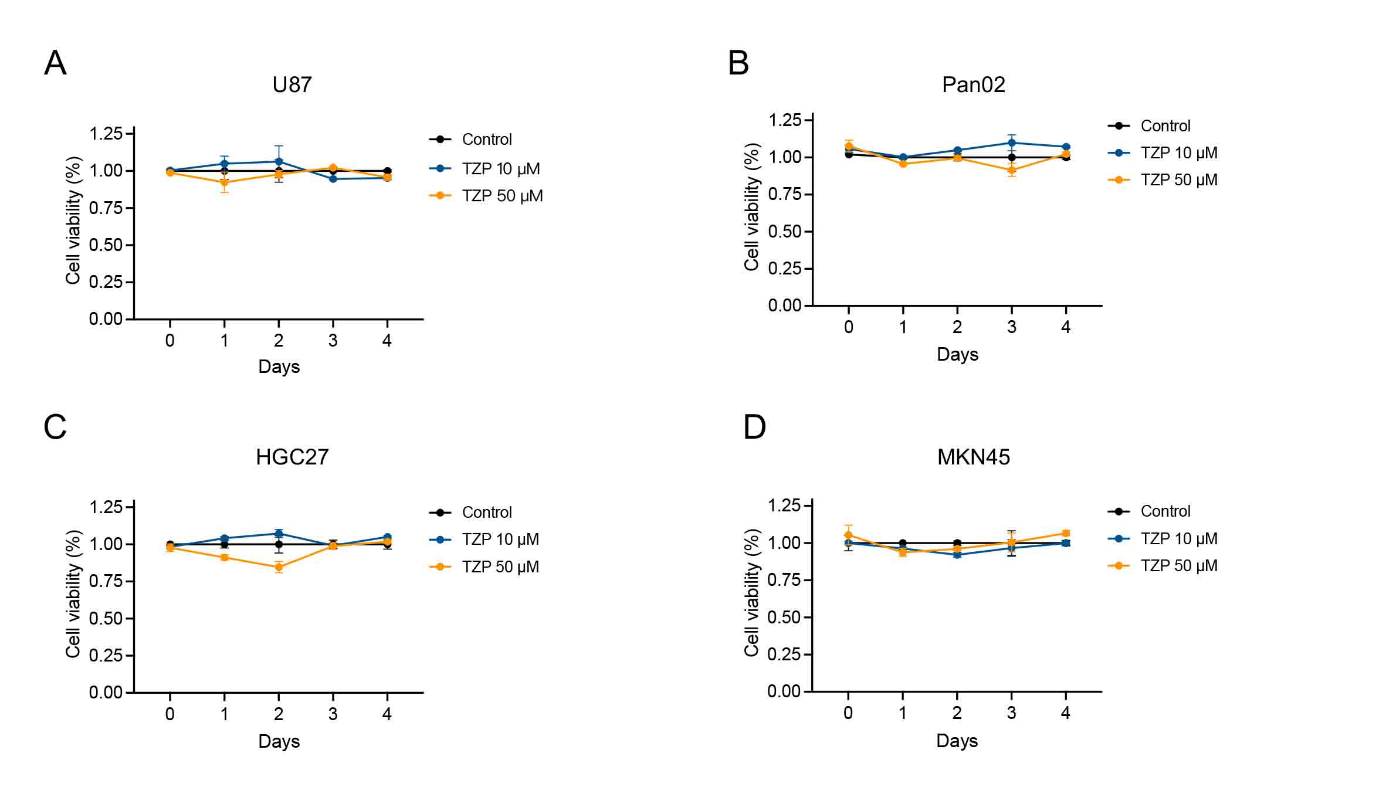


**Figure S3. The effect of TZP on viability of other tumor cell lines.**

The cell proliferation of glioma cell line (U87MG), pancreas-02 (Pan02), human gastric cancer cells (HGC27 and MKN45) and Matsuo-Kaneda-Nakamura Number 45 (MKN45 cells) were detected by the CCK-8 assay after TZP treatment for 48 h (10 and 50 μM). Data were shown as mean±SEM. One-way ANOVA was performed.


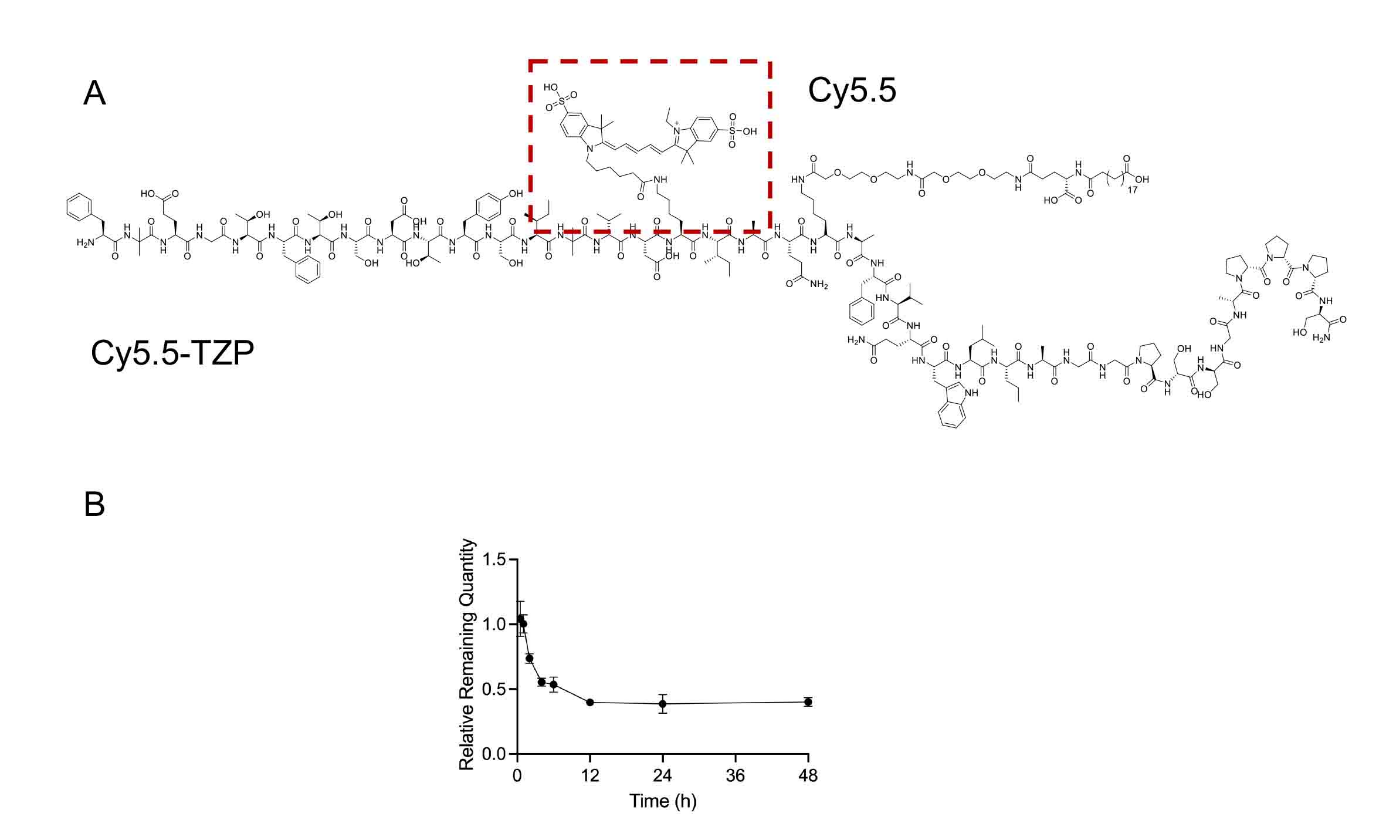


**Figure S4:** **Synthesis of Cy5.5-TZP and detection of half-life after intraperitoneal injection of Cy5.5-TZP in mice.**

(A) Chemical formulas and equations for Cy5.5-TZP. (B) The plasma concentration-time curve was obtained by multimode microplate reader.


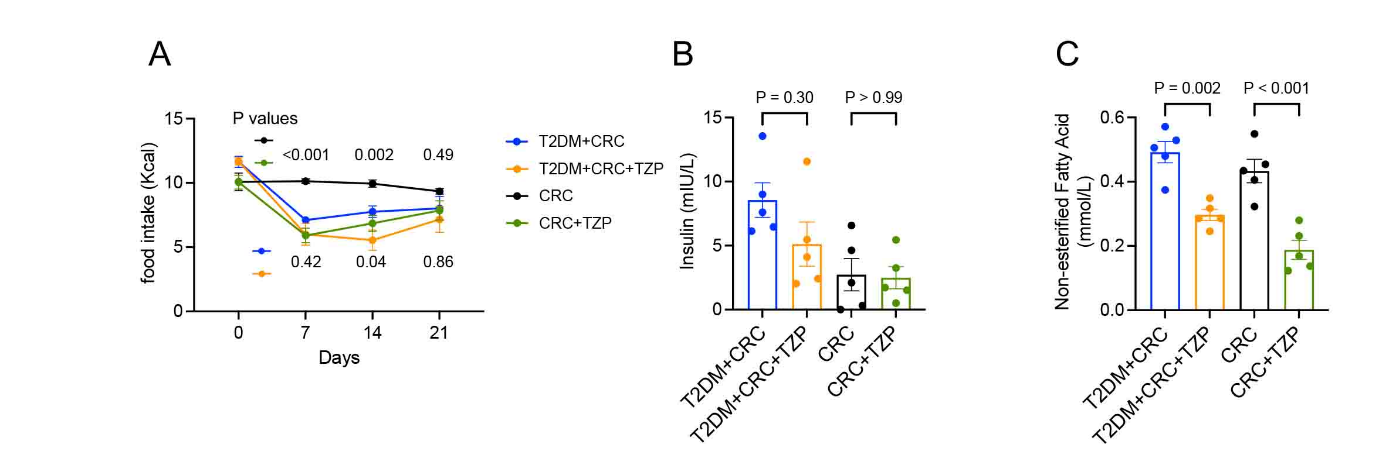


**Figure S5. Effects of TZP on feed intake, serum insulin and serum fatty acid in a T2DM mice with colorectal cancer.**

(A) Weekly energy intake, calculated by gram weight of diet consumed×caloric content of diet, in chow and 60% HFD fed WT and T2DM mice with CRC (n=5-8). (B)The insulin levels in serum samples were determined by immunoassay (n=5). (C) Serum non-esterified fatty acids were detected by enzymatical assay (n=5). Data were shown as mean±SEM. One-way ANOVA was performed, and P values were adjusted with the Tukey multiple comparisons test (A-C).


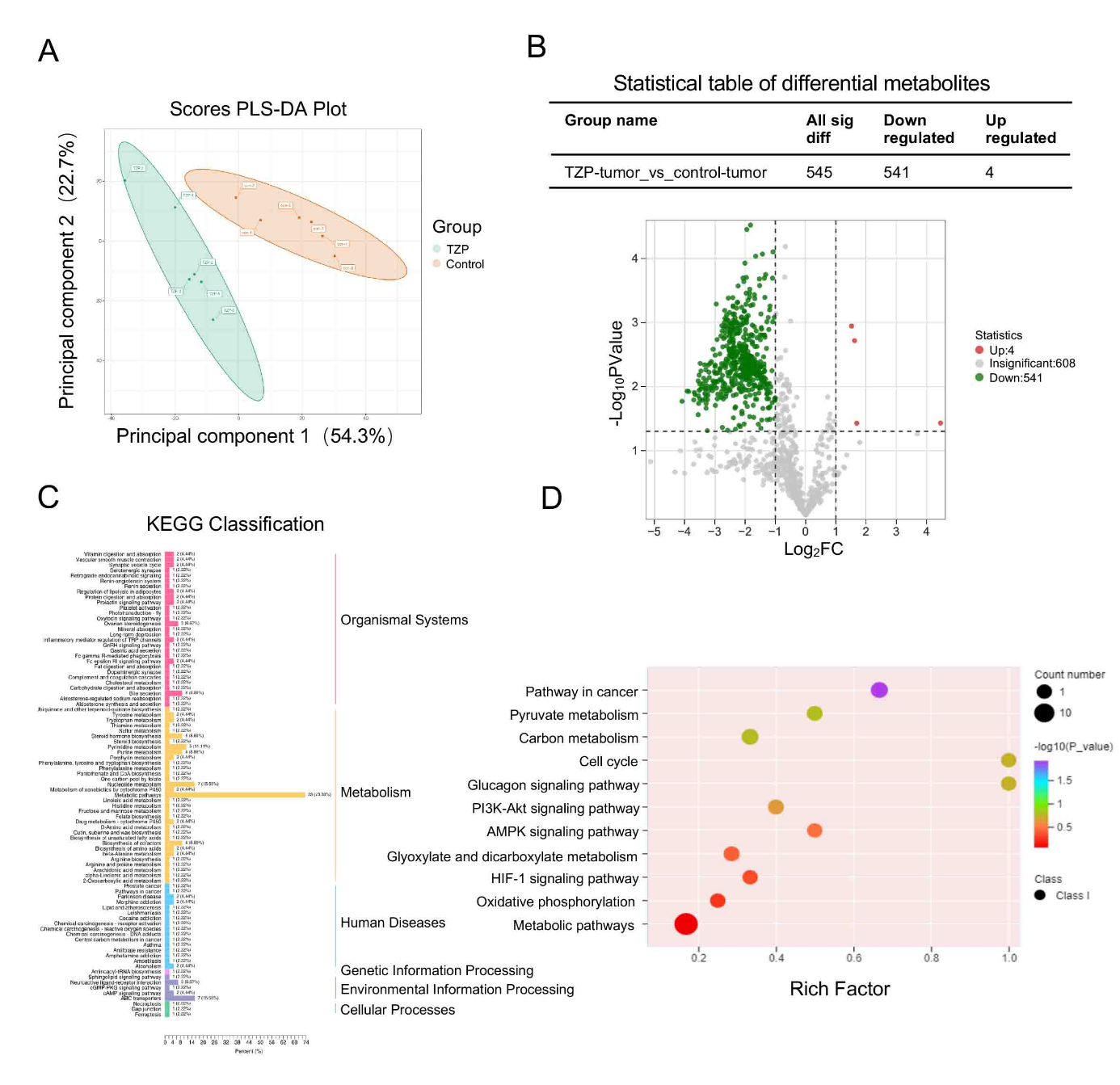


**Figure S6.** **Spatial metabolomics revealed the differences in metabolic products and pathway alterations in tumor tissues between the TZP treated and control groups.**

(A) Multivariate analysis of metabolites in control and TZP-treated samples was performed by partial least squares discriminant analysis (PLS-DA). (B)Volcanic map of tumor metabolites in control and TZP treated mice. Red dots on the volcano map indicated upregulation and green dots, downregulation. (C)The annotation results of the significantly different metabolites were classified according to the type of pathways in KEGG. The ordinate was the name of the KEGG metabolic pathway, and the abscess was the number of differential metabolites annotated to the pathway and their proportion to the total number of differential metabolites annotated. (D) Bubble diagram of the KEGG pathway annotation. The x-axis indicated the pathway impact factor and the y-axis, the pathway term. Dot color indicated the q value and dot size, the counts of metabolites.


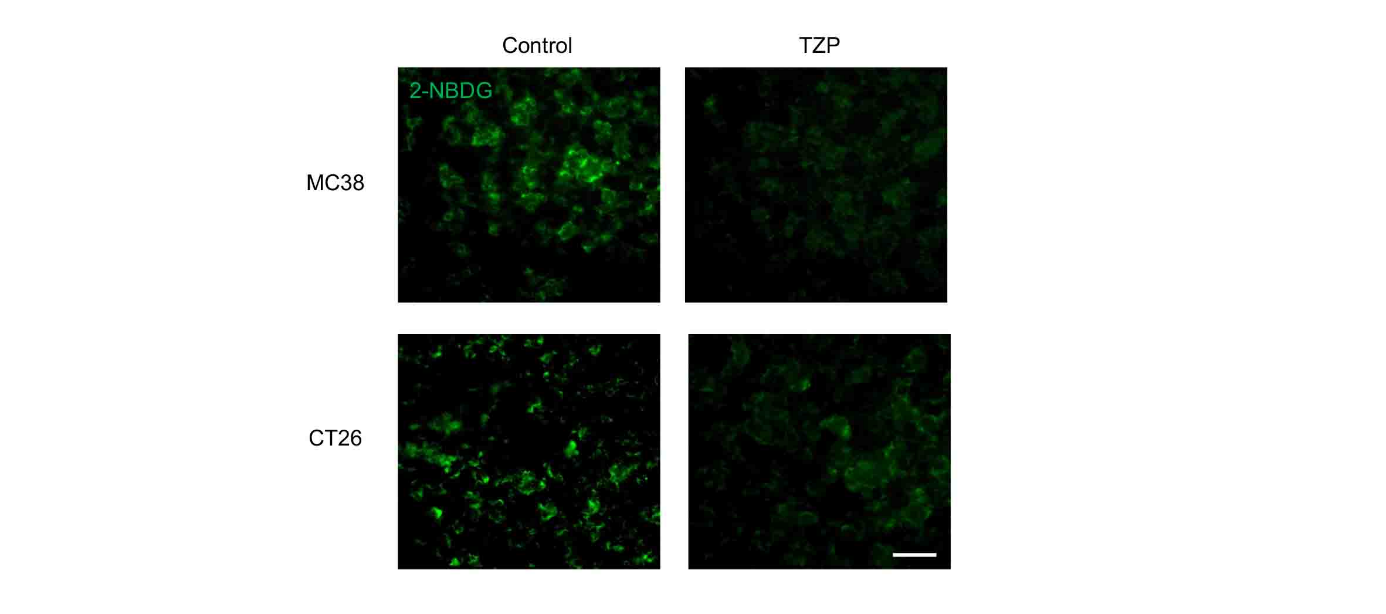


**Figure S7: Detection of glucose uptake in MC38 and CT26 cells.**

Uptake of 2-(N-(7-nitrobenz-2-oxa-1,3-diazol-4-yl) amino)-2-deoxyglucose (2-NBDG) was determined by immunofluorescence staining (Scale bar: 100 μm).


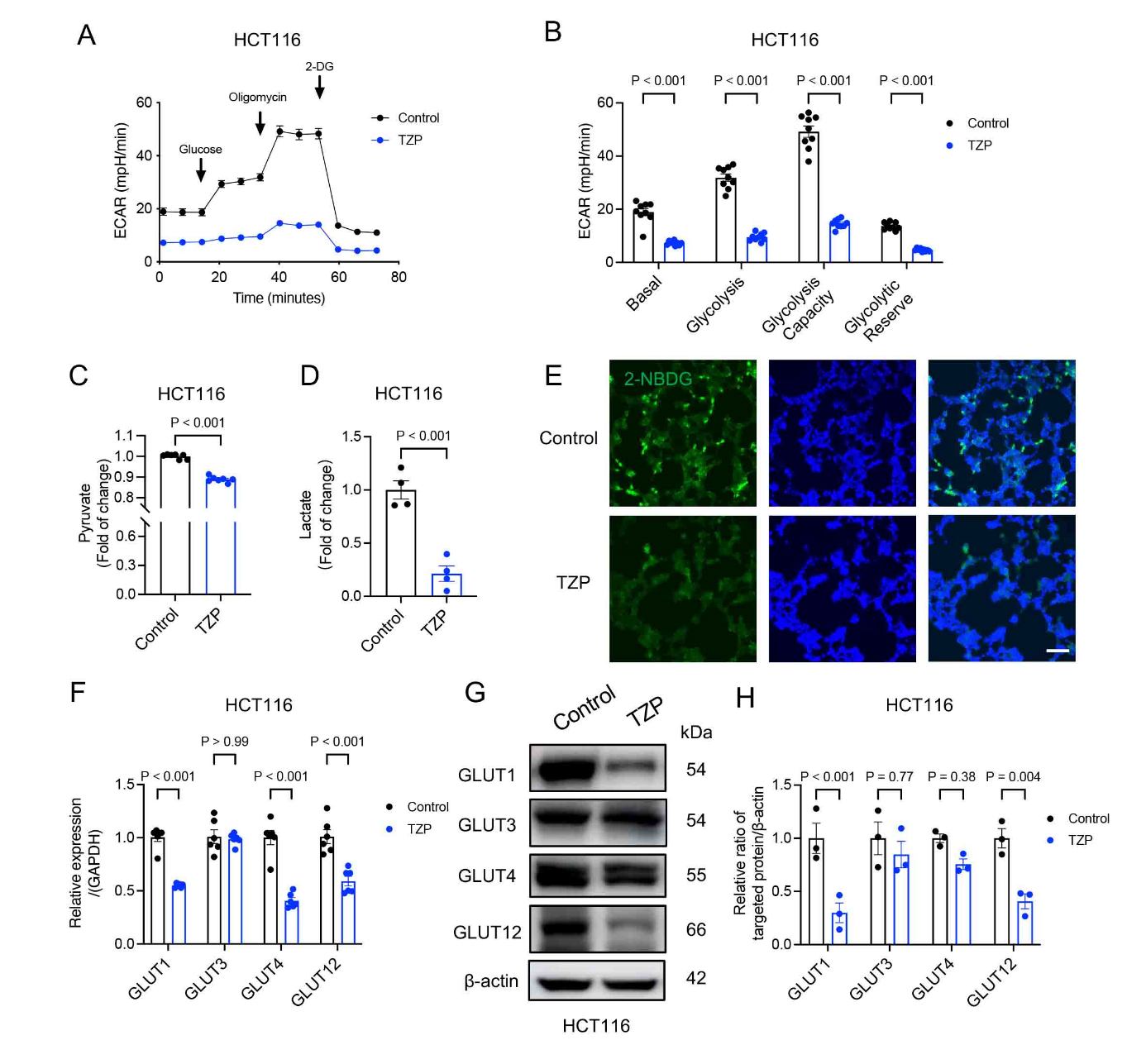


**Figure S8.** **The effect of tirzepatide on the glycolytic levels and expression of glucose transporters in HCT116 cells.**

HCT116 cells were divided into two groups (Control vs TZP). (A-B) Glycolytic flux (extracellular acidification rate [ECAR] using Seahorse XF) was measured in HCT116 cells (n=9 per time point). Representative basal respiration, glycolysis, glycolysis capacity and glycolytic reserve were shown, and ECAR, quantified (n=9). (C-D) Quantification of pyruvate and lactate production in HCT116 cells. (E) Uptake of 2-NBDG was determined by immunofluorescence staining. (F) mRNA changes were validated by qPCR of selected glucose transporters genes in HCT116. (G) Western blotting detected expression of GLUT1, GLUT3, GLUT4 and GLUT12. (H) Ratio of GLUT1, GLUT3, GLUT4 and GLUT12 to β-actin. Data were shown as mean±SEM. Statistical significance was determined by two-way repeated measurement ANOVA with Tukey multiple comparisons test (B, F, H) and unpaired t test (C, D).


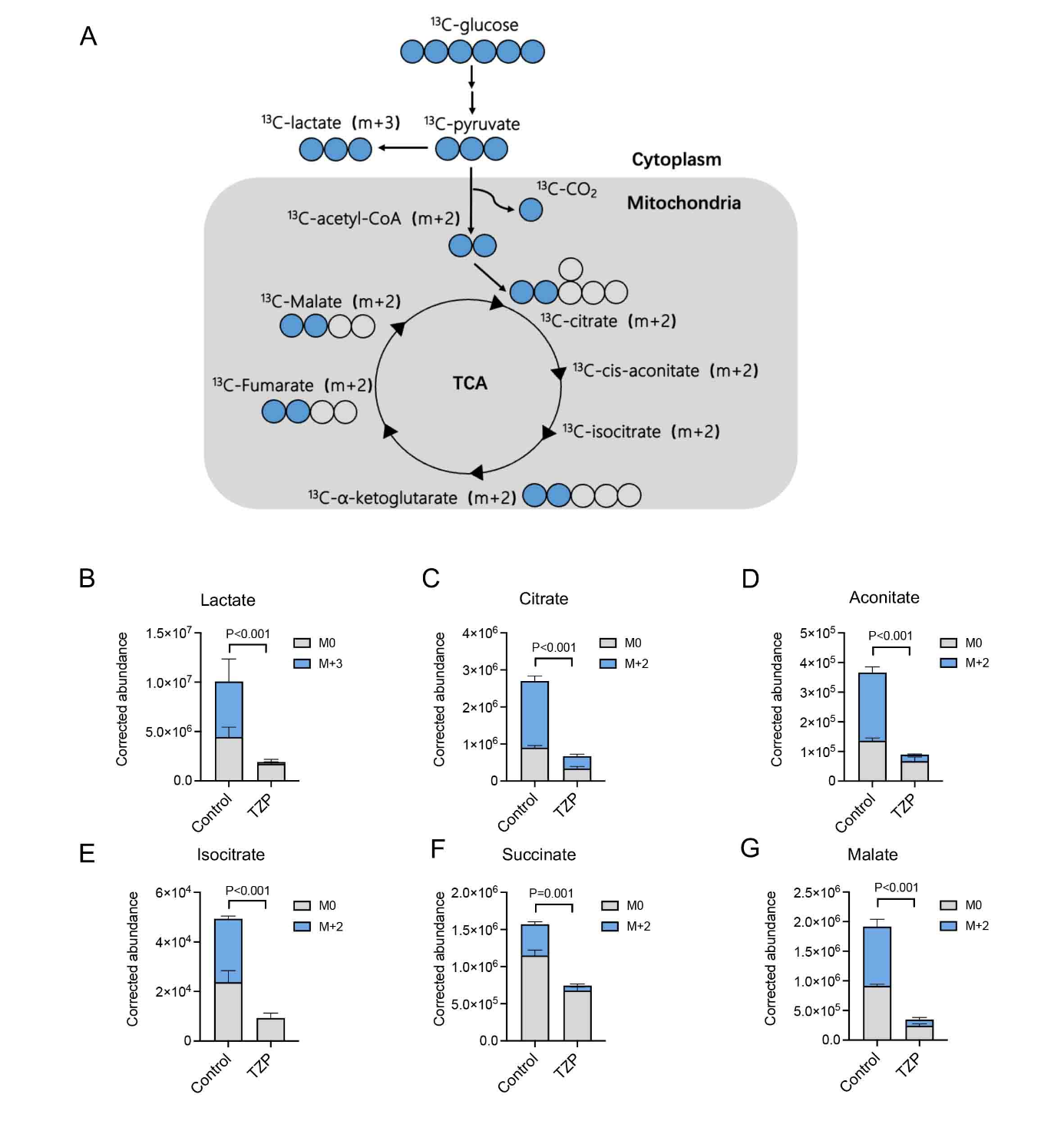


**Figure S9.** **Determination of metabolic intermediates in the TCA cycle using isotope labeling in HCT116 cells treated with TZP.**

(A) Schematic diagram of the labeling of [U-^13^C6] glucose carbon in glycolysis and the first round of the TCA cycle. Labeled carbons were shown in blue, and unlabeled, in grey. (B-G) The ^13^C isotopic enrichments of lactate, citrate, aconitate, isocitrate, succinate and malate. Data were shown as mean±SEM. Statistical significance was determined by unpaired t test (B-G).


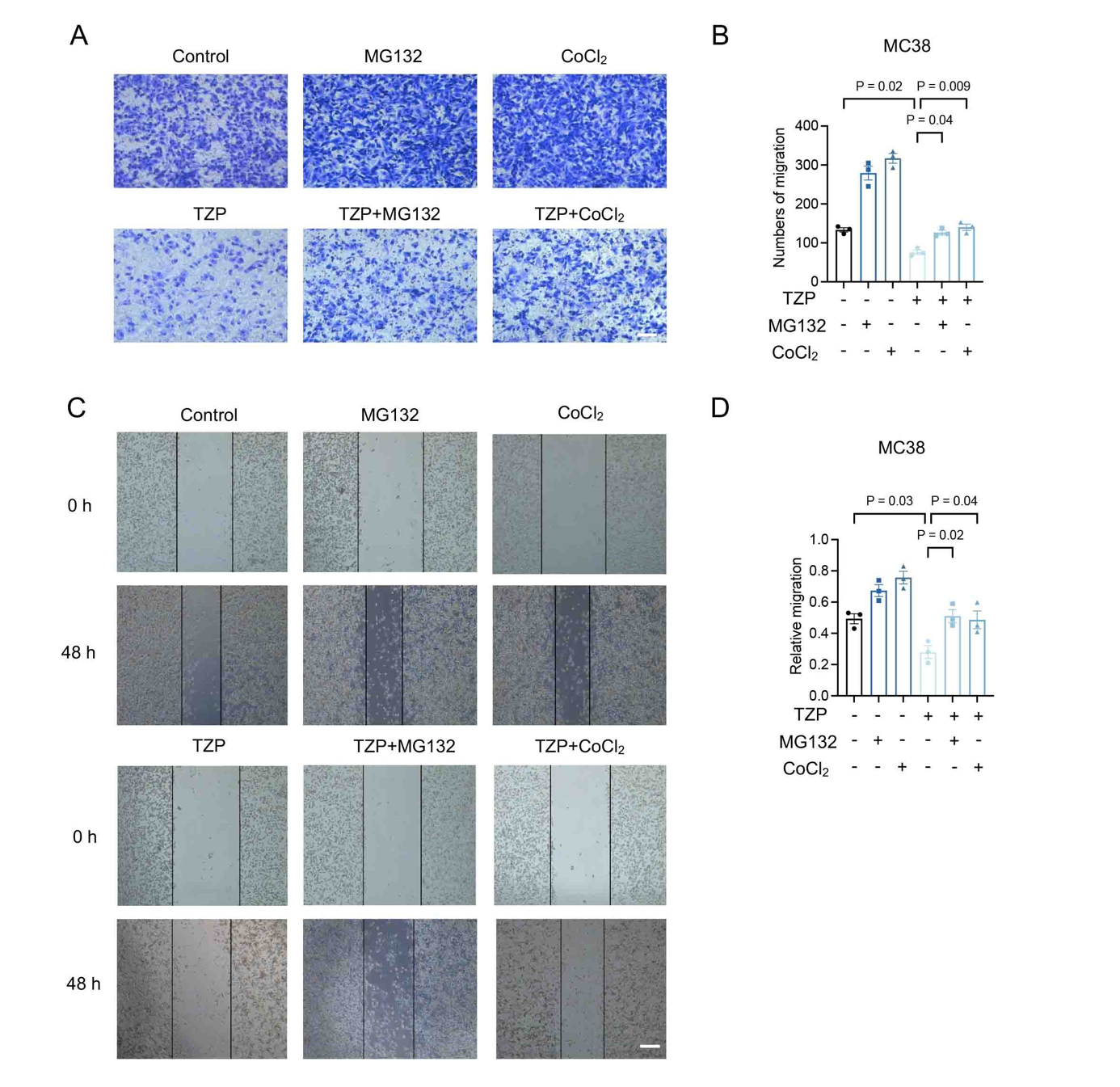


**Figure S10. Detection of cell migration in transwells and by wound healing in MC38 cells with increased HIF-1α stability in response to TZP treatment.**

MC38 cells were cultured with CoCl_2_ (500 µM) for 6 h to induce the hypoxia or treated with MG132 (5 µM), a proteasome inhibitor, for 6 h and then incubated with or without TZP (50 µM). (A) Migration/invasion was evaluated by the transwell assay (n=3) (Scale bar: 200 μm). (C-D) Migration was assessed by the wound healing assay (n=3) (Scale bar: 200 μm). Data were shown as mean±SEM. Statistical significance was determined by one-way repeated measurement ANOVA with Tukey multiple comparisons test (B, D)


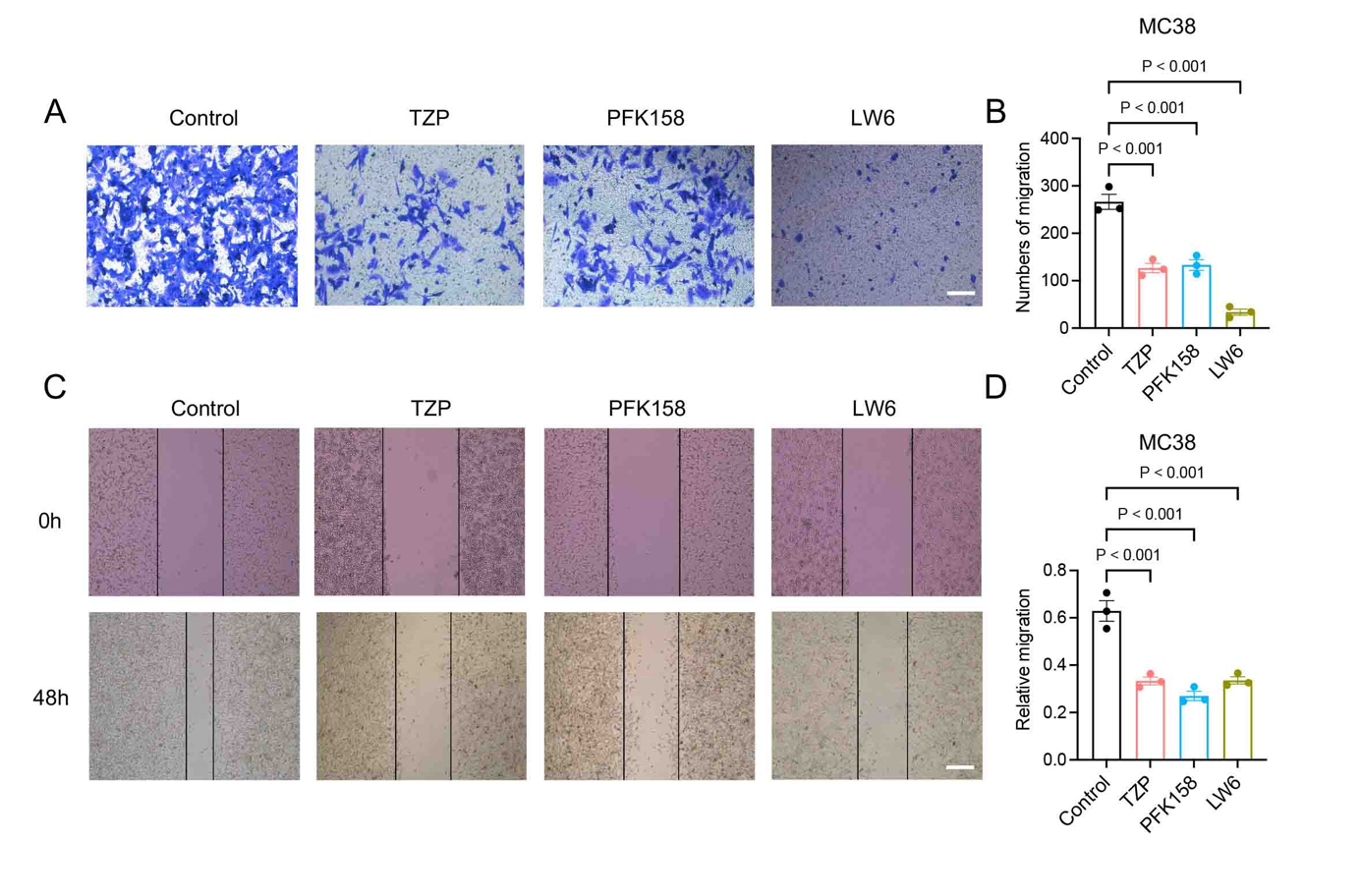


**Figure S11. Detection of cell migration in transwell and wound healing assays in MC38 cells treated with TZP as compared with chemical inhibition of HIF-1α and PKFKB3.**

MC38 cells were cultured with chemical inhibitors of PFKFB3 (PFK-158, 10 μM) or HIF-1α (LW6, 50 μM) or treated with TZP for 48 h to detecte cells migration. (A-B) Migration/invasion was evaluated by the transwell assay (n=3) (Scale bar: 200 μm). (C-D) Migration was assessed by the wound healing assay (n=3) (Scale bar: 200 μm). Data were shown as mean±SEM. Statistical significance was determined by one-way repeated measurement ANOVA with Tukey multiple comparisons test (B, D)


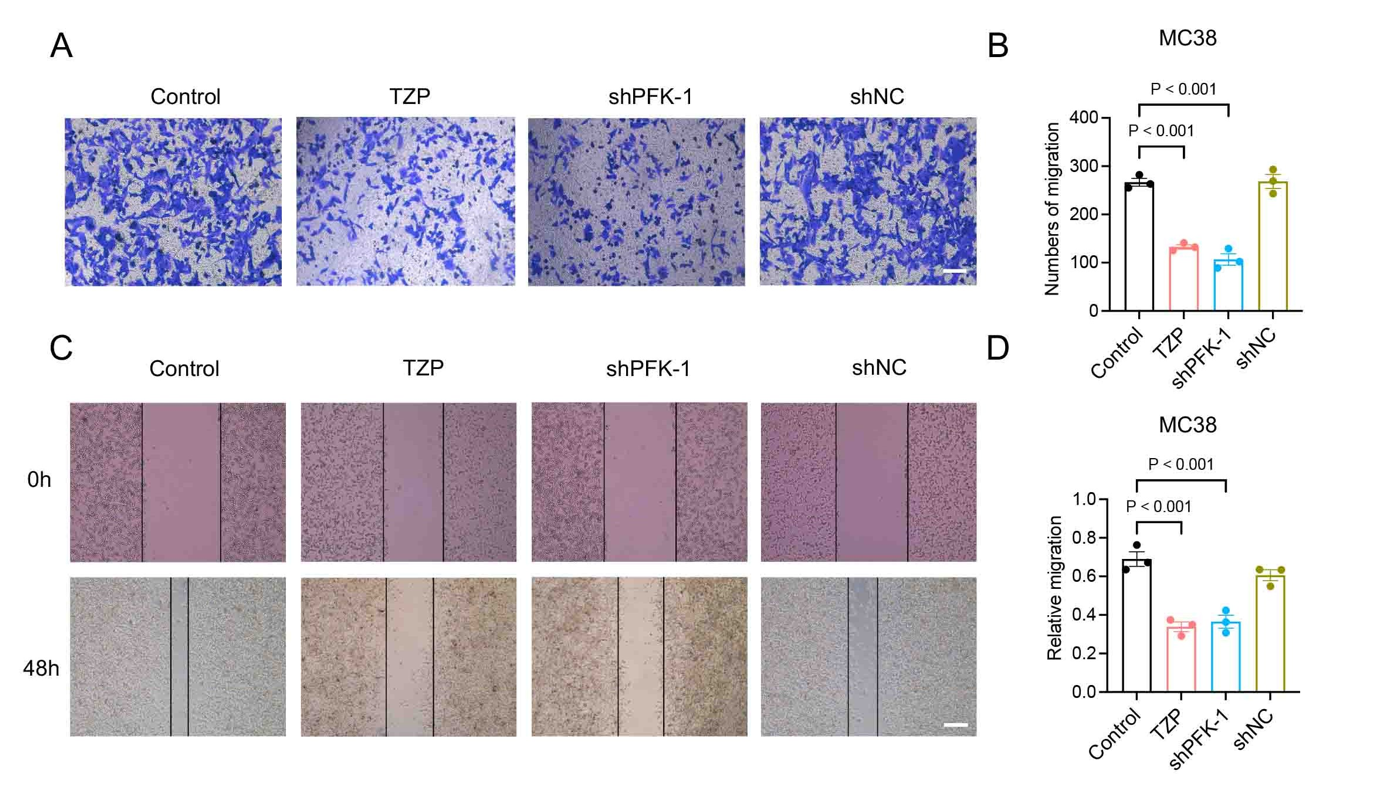


**Figure S12. Detection of cell migration by transwell and wound healing assay in MC38 cells treated with TZP as compared to lentivirus-mediated PFK-1 downregulation.**

MC38 cells were infected with the lentivirus containing selective shPFK-1 for 12 h or treated with TZP for 48 h to detecte cells migration. (A-B) Migration/invasion was evaluated by the transwell assay (n=3) (Scale bar: 200 μm). (C-D) Migration was assessed by the wound healing assay (n=3) (Scale bar: 200 μm). Data were shown as mean±SEM. Statistical significance was determined by one-way repeated measurement ANOVA with Tukey multiple comparisons test (B, D)

**
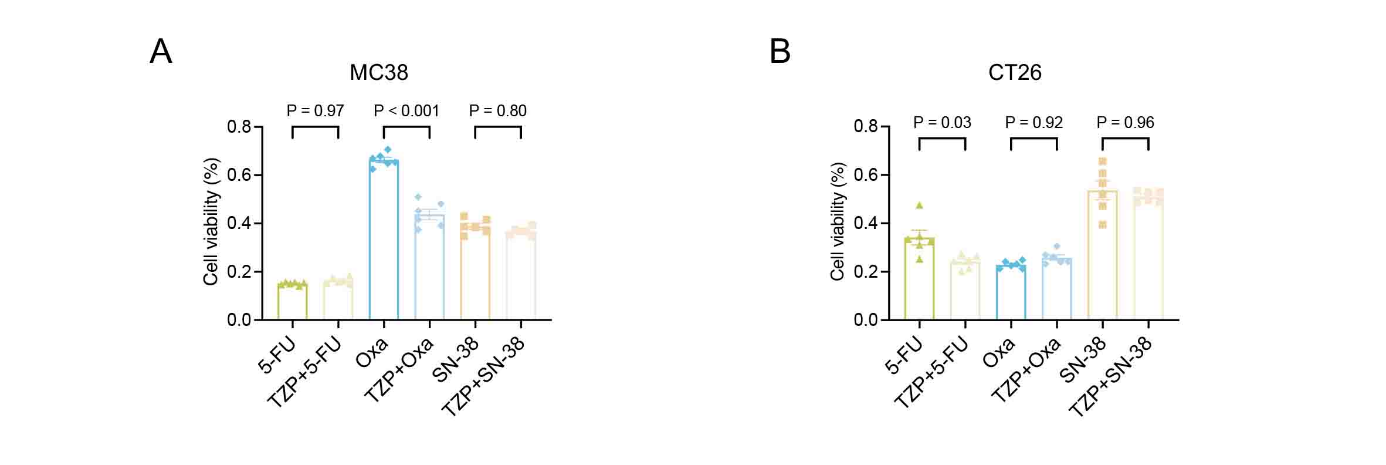
**

**Figure S13.** **Detection of the synergistic effect of TZP and anticancer drugs.**

MC38 and CT26 cells were treated with TZP combined with 5-FU (5-Fluorouracil), SN-38 (Irinotecan) or Oxa (Oxaliplatin). Cell viability was detected by CCK-8 assay. Data were shown as mean±SEM. Statistical significance was determined by one-way repeated measures ANOVA with Tukey multiple comparisons test.


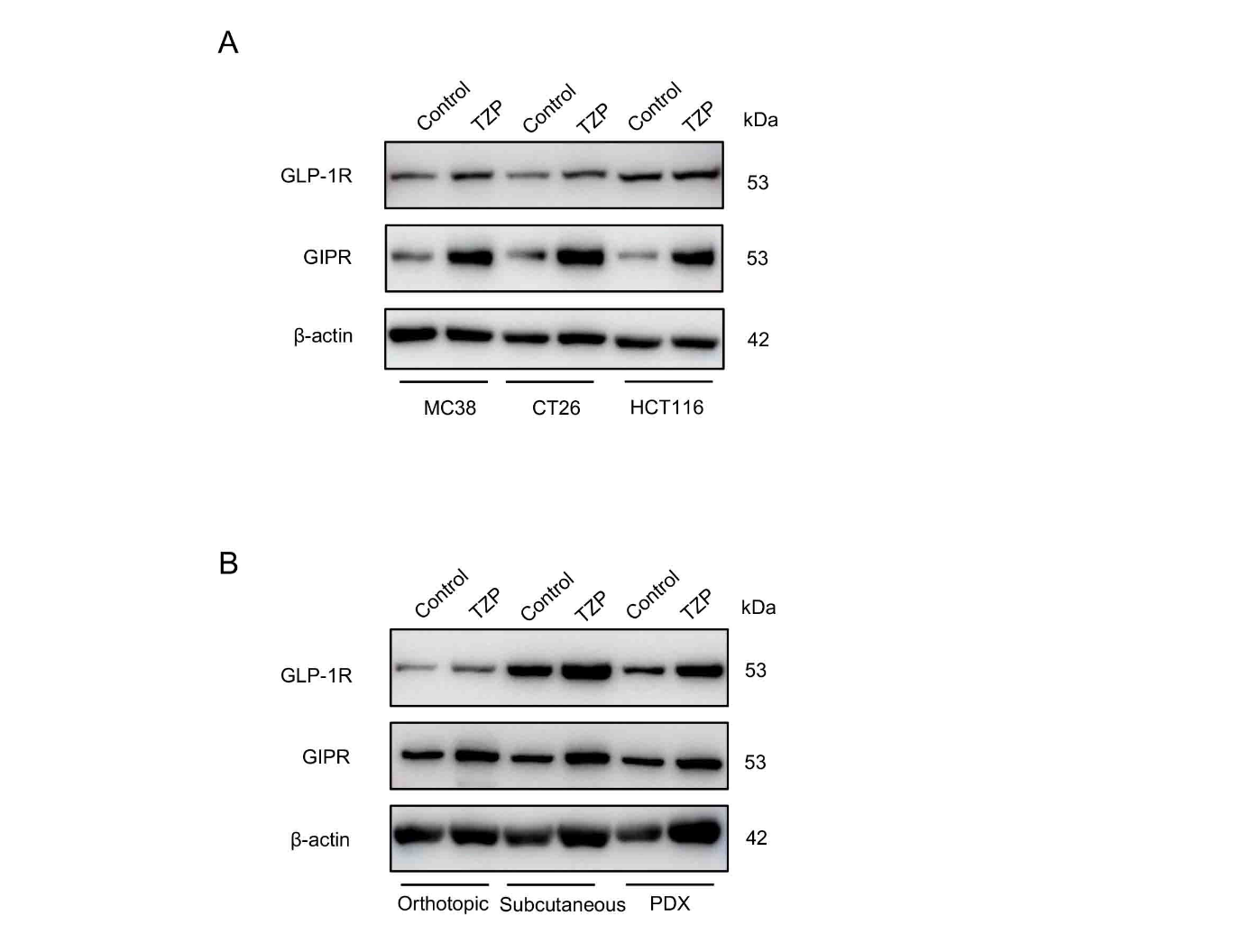


**Figure S14.** **Effects of TZP on expression of GLP-1R and GIPR in various CRC cell lines and CRC tumor tissues.**

(A) CRC cells were incubated at the presence or absence of TZP (50 μM) for 48 h and the extracts were fractionated by SDS-PAGE and analyzed by western blotting with GIPR and GLP-1R. β-actin was used as protein loading control. (B) The orthotopic, subcutaneous and PDX tumors from the *in vivo* experimental groups were lysed and used for western blotting analysis with GIPR and GLP-1R. β-actin was used as protein loading control.

| Clone Name | Sequence | Source / Repository |
| --- | --- | --- |
| PFKFB3 | F: 5ʹ-TCA TCG AGT CGG TCT GTG ACG A-3ʹ | Mouse |
|  | R: 5ʹ-CAT GGC TTC TGC TGA GTT GCA G-3ʹ |  |
| PFKM | F: 5ʹ-TGG CAC AGT GAT TGG AAG TG-3ʹ | Mouse |
|  | R: 5ʹ-GCT CCA CTC TGA ACG GAA AG-3ʹ |  |
| PFKP | F: 5ʹ-AGG AGG GCA AAG GAG TGT TT-3ʹ | Mouse |
|  | R: 5ʹ-TTG GCA GAA ATC TTG GTT CC-3ʹ |  |
| ALDOA | F: 5’-CAG GAA AGC AAC TGC CAC CGG CAC -3ʹ | Mouse |
|  | R: 5’-GGA TTC ACA CGG TCG TCT GCA GTC-3ʹ |  |
| LDHA | F: 5’-TGT CTC CAG CAA AGA CTA CTG T-3ʹ | Mouse |
|  | R: 5’-GAC TGT ACT TGA CAA TGT TGG GA -3ʹ |  |
| GPI1 | F: 5’-CTC AAG CTG CGC GAA CTT TTT-3ʹ | Mouse |
|  | R: 5’-GGT TCT TGG AGT AGT CCA CCA G-3ʹ |  |
| HK2 | F: 5ʹ-CTA AGG GGT TCA AGT CCA GTG G-3ʹ | Mouse |
|  | R: 5ʹ-AGA CCA ATC TCG CAG TTC TGA-3ʹ |  |
| HK1 | F: 5ʹ-CAA GAA ATT ACC CGT GGG ATT CA-3ʹ | Mouse |
|  | R: 5ʹ-CAA TGT TAG CGT CAT AGT CCC C-3ʹ |  |

**Table S1. List of primer sequences used for quantitative RT-PCR for** **glycolysis-related genes.**

| Clone Name | Sequence | Source / Repository |
| --- | --- | --- |
| GLUT1 | F: 5ʹ-TCGTCGGCATCCTCATCGCC-3ʹ | Human |
|  | R: 5ʹ-CCGGTTCTCCTCGTTGCGGT-3ʹ |  |
| GLUT3 | F: 5ʹ-GCTGGGCATCGTTGTTGGA-3ʹ | Human |
|  | R: 5ʹ-GCACTTTGTAGGATAGCAGGAAG-3ʹ |  |
| GLUT4 | F: 5ʹ-GCCATGAGCTACGTCTCCATT-3ʹ | Human |
|  | R: 5ʹ-GGCCACGATGAACCAAGGAA-3ʹ |  |
| GLUT12 | F: 5’-AACATGCGGACCCGAATAATG-3ʹ | Human |
|  | R: 5’-AATGACCTTGACGACTCCAAC-3ʹ |  |

**Table S2. List of primer sequences used for quantitative RT-PCR for glucose transporter genes.**
